# Polybrominated Diphenyl Ethers and Heavy Metals in a Regulated E-Waste Recycling Site, Eastern China: Implications for Risk Management

**DOI:** 10.3390/molecules26082169

**Published:** 2021-04-09

**Authors:** Hongmin Yin, Jiayi Ma, Zhidong Li, Yonghong Li, Tong Meng, Zhenwu Tang

**Affiliations:** 1 College of Environmental Science and Engineering, North China Electric Power University, Beijing 102206, China; hongminyin@ncepu.edu.cn (H.Y.); mengtong@ncepu.edu.cn (T.M.); 2 College of Life and Environmental Sciences, Minzu University of China, Beijing 100081, China; 17010176@muc.edu.cn (J.M.); 20400254@muc.edu.cn (Y.L.); 3 Cangzhou Ecology and Environment Bureau, Cangzhou 061000, China; lzdhdm@163.com

**Keywords:** regulated e-waste recycling, polybrominated diphenyl ethers (PBDEs), heavy metals, environmental media, vegetable, risks

## Abstract

Serious pollution of multiple chemicals in irregulated e-waste recycling sites (IR-sites) were extensively investigated. However, little is known about the pollution in regulated sites. This study investigated the occurrence of 21 polybrominated diphenyl ethers (PBDEs) and 10 metals in a regulated site, in Eastern China. The concentrations of PBDEs and Cd, Cu, Pb, Sb, and Zn in soils and sediments were 1–4 and 1–3 orders of magnitude lower than those reported in the IR-sites, respectively. However, these were generally comparable to those in the urban and industrial areas. In general, a moderate pollution of PBDEs and metals was present in the vegetables in this area. A health risk assessment model was used to calculate human exposure to metals in soils. The summed non-carcinogenic risks of metals and PBDEs in the investigated soils were 1.59–3.27 and 0.25–0.51 for children and adults, respectively. Arsenic contributed to 47% of the total risks and As risks in 71.4% of the total soil samples exceeded the acceptable level. These results suggested that the pollution from e-waste recycling could be substantially decreased by the regulated activities, relative to poorly controlled operations, but arsenic pollution from the regulated cycling should be further controlled.

## 1. Introduction

The amount of waste of electronic and electrical equipment is continually increasing with the widespread use of electronic products. The Global E-waste Monitor reported that the world generation of electronic waste (e-waste) reached 41.8, 44.7, and 53.6 million Mt in 2014, 2016, and 2019, respectively; and it is expected to grow to 74.7 million tons by 2030 [1,2,3]. Correspondingly, the e-waste from Asia, Americas, Europe, and Africa in 2019 were at 24.9, 13.1, 12.0, and 2.90 Mt, respectively [3]. However, only 15.6% to 20.0% of e-waste was documented to be collected and properly recycled in 2014, 2016, and 2019 around the world [1,2,3]. Therefore, the safety of the recycling of e-waste is of increasing concern.

E-waste usually contains or is contaminated with the multiclass hazardous chemicals. Typically, high concentrations of lead, cadmium, and mercury are often observed in e-wastes due to these metals used widely in battery [4]. Some lead compounds are also used as a stabilizer or plasticizer [5,6]. Chromium was used in data tapes, floppy disks, and are usually used as pigments in many electrical and electronic products [5,7]. Copper is frequently used in printed circuit boards [8,9]. Antimony trioxide is used as flame retardants [10,11]. In addition, many organic chemicals are also used widely in electrical and electronic products as flame retardants. For example, polybrominated diphenyl ethers (PBDEs) and dechlorane plus (DPs) are mainly used in cables. DPs are usually used in connector coating and electric wires, and PBDEs are usually found in printed circuit boards [12,13]. Additionally, polychlorinated biphenyls (PCBs) are used in transformers and capacitors [14]. These chemicals are easily released into the environment during the dismantling or treatment of e-waste and can cause adverse effects on the human body [15,16,17,18]. Meanwhile, many kinds of toxic pollutants such as polycyclic aromatic hydrocarbons (PAHs) and polychlorinated dibenzo-*p*-dioxins and dibenzofurans could be produced in the process of e-waste incineration [19].

In the past decades, large amounts of e-waste were transported to developing countries for recycling. Typically, about 70% of the world’s e-waste were processed in China in 2012 [20]. Due to the lack of related technology and cost savings, e-waste was recycled in many countries by the primary method, inducing manual dismantling, crude processing of circuit board, acid treatment to recover metals, open burning, and dumping of residues [21,22]. This led to many crude e-waste recycling sites formed in many low-income countries, such as India, China, Ghana, and Nigeria [15,23,24,25]. In China, the irregulated e-waste recycling sites were mainly concentrated in family workshops in Shantou, Qingyuan, and Taizhou. Mechanical recovery dominated in these irregulated process, which was mainly to extract precious metals. Many previous surveys found that serious environmental pollution occurs in these sites. Labunska et al. reported that the PBDE concentrations reached 390 µg·g^−1^ in soils, from an e-waste open-burning area, and reached 220 µg·g^−1^ in sediments surrounding a circuit board shredding in the Guiyu town, Southern China [26]. Moeckel et al. also reported that PAH concentrations caused by e-waste dumping activities were up to 16.0 µg·g^−1^ in soils from Agbogbloshie, Ghana [27]. Meanwhile, Wu et al. found that the mean concentrations (µg·g^−1^) of Sb, Sn, and Ag reached 5,003, 2,527, and 47.9, respectively, in surface soils, at an e-waste burnt site in Guiyu, China [28]. The serious environmental pollutions could directly cause high levels of human internal exposure to these chemicals, likely resulting in high risks to human health [29,30].

In recent years, the irregulated e-waste recycling was gradually banned and relevant policies and technical regulations were developed and implemented in many countries [3,31]. The e-waste recycling operations is usually concentrated to industrial parks and many pollution-control measures are applied to recycling processes [32]. Currently, many venous industrial parks are established in China to concentrate the recycling activities of e-waste [21]. Many pollution-control measures, including safe storage, closely mechanical dismantling, and negative pressure to control dismantling dust, are performed in the recycling processes [21]. These measures are generally considered to be effective in reducing chemical emissions. However, there are few systematic investigations of the environment contamination surrounding the regulated recycling sites and the associated human health risks remain unknown.

In this study, we used a regulated e-waste recycling site in Eastern China, as a case study. Our main aim was to characterize the concentrations and risks of PBDEs and metals, two characteristic pollutants in the e-waste recycling process, in the soils, sediments and the vegetables surrounding the site. Then, the contamination levels and the risks posed by these chemicals in this site and in other areas, and in particular in irregulated e-waste recycling sites, were compared, in order to understand if the current pollution-control measures in the regulated e-waste recycling are effective and safe. Our results will provide important insight into these chemicals in regulated e-waste recycling area, and might be useful in informing the development of risk management measures.

## 2. Results and Discussion

### 2.1. Contamination of PBDEs

The concentrations of PBDEs in soil, sediment, and vegetable samples are summarized in Table 1. In the soil samples, the detection frequency of BDE-209 was highest (100%), followed by BDE-154 (33%). However, the detection frequencies of most other congeners were less than 20%. The concentrations of BDE-209 and Σ_21_PBDEs were 3.05–1331 and 3.05–1366 ng·g^−1^, respectively. Higher concentrations (ng·g^−1^) of Σ_21_PBDEs were observed in the sites S_19_ (1366) and S_20_ (593) (Figure S1 from Supplementary Materials). The e-waste was piled up at the two sampling sites. BDE-209 accounted for 94.7% of the concentration of Σ_21_PBDEs. The octa-BDEs (including BDE-196, -197, -201, -202, -203, and BDE-205) and the nona-BDEs (including BDE-206, -207, and BDE-208) represented 1.86% and 2.16% of the concentrations of Σ_21_PBDEs, respectively. The results reflected that the e-waste recycled in the site might originate mainly from China. Many previous studies confirmed that deca-BDE predominated in the e-waste from China [33,34,35].

In the sediment samples, the detection frequencies of BDE-209 and BDE-154 were 100% and 57.1%, respectively. Low detection frequencies of the other congeners were also observed. The concentrations of BDE-209 and Σ_21_PBDEs in sediments were in the range of 4.27–314 and 4.31–327 ng·g^−1^, respectively. Higher concentrations of Σ_21_PBDEs were found in the sites S_24_ and S_25_. BDE-209 represented 93.2% of the Σ_21_PBDE concentrations. In general, the contributions of other individual congeners were less than 1%.

The detection frequencies of individual congeners in the vegetable samples were different from those of the soil and sediment samples. BDE-28, -47, -66, -99, -100, -154, -183, -206, -207, -208, and BDE-209 were detectable in more than 50% of vegetable samples, likely reflecting the stronger bioaccumulation of these chemicals. The median concentrations of BDE-209 and Σ_21_PBDEs in vegetable samples were 201 and 204 ng·g^−1^, respectively. The highest Σ_21_PBDE concentration was found in the baby’s breath samples at site V_7_ (425 ng·g^−1^ dry weight), a site near the soil sampling site S_20_. Interestingly, the congener profiles of PBDEs in vegetables were similar to those in soils and sediments, although the detection frequencies of congeners differed, suggesting the vegetable PBDEs mainly originated from the emission from the recycling operations in the site.

**Figure 1:**
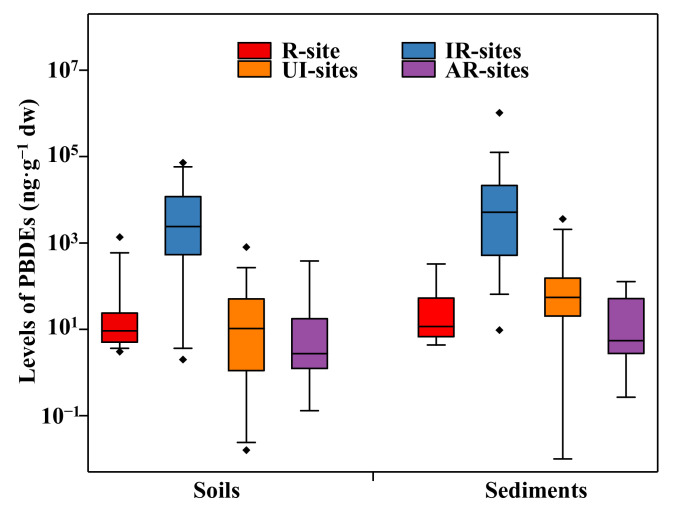
Comparison of the PBDE concentrations (ng·g^−1^ dry weight) in soils and sediments in the study area with the other areas. R-site, regulated e-waste recycling site (this study); IR-sites, irregulated e-waste recycling sites; UI-areas, urban and industrial areas; AR-areas, agricultural and rural areas (data from the Tables S3 and S4 from Supplementary Materials) The maximum, minimum, and median (or mean) of PBDE concentrations in each literature were selected as a total sample. The horizontal lines represent the 10th, 50th, and 90th percentiles, and the boxes represent the 25th and 75th percentiles; the asterisk below or above indicates outliers.

The PBDE concentrations found in this study were compared to those observed from the IR-sites, UI-areas, and the AR-areas (Tables S3–S5 from Supplementary Materials). The median concentrations of PBDEs of soils and sediments in our study were 1–4 orders of magnitude lower than those separately reported in some IR-sites in China (Figure 1). In particular, the maximum concentration of total PBDEs in the soils from an IR-site in Guiyu was one order of magnitude higher than that in this area [36]. Huang et al. [37] reported that the maximum concentration of total PBDEs in the sediment in an IR-site reached 1030 µg·g^−1^, four orders of magnitude higher than our result. For the vegetables, however, the concentrations of PBDEs found in this study were generally higher than those reported in the IR-sites. In this study, most vegetable samples were collected within the recycling plant. In most IR-sites selected, almost all vegetable samples were collected far from the recycling plant [38,39]. In addition, the differences of plant species and their growth period among different studies might also be responsible for the comparison results.

The contamination levels of PBDEs in this site were compared with those in the UI-areas. Median concentrations of PBDEs of soils and sediments in this site were comparable to or lower than those in the UI-areas (Figure 1). Typically, the median concentrations of soil PBDEs from an industrial area in Tianjin and an urban area in Shenyang-Fushun, China, were similar to those reported in this study [40,41]. The concentrations of sediment PBDEs in this study were generally lower than those reported in UI-areas. Even, the concentrations of total PBDEs in some urban rivers in the Yangtze River Delta and some urban reaches of the Pearl River were one order of magnitude higher than those observed in this area [42,43]. The concentrations of total PBDEs in vegetable samples observed in this study were lower than those reported in pine needle from Shanghai and camphor bark from the Jiangsu Province, China [44,45]. However, our results were one to two orders of magnitude higher than those observed in the UI-areas in Inner Mongolia and in the Zhejiang Province, China [46,47].

The PBDE concentrations observed in this study were also compared with those reported in some agricultural regions in China (Figure 1). In general, the median PBDE concentrations in soils and sediments from our study were 6.36 and 2.12 times higher than those in the AR-areas (Tables S3 and S4 from Supplementary Materials). However, the median PBDE concentrations of soils in Shanghai and sediments in the Chaohu Lake were one order of magnitude higher than those in our study [48,49]. This was likely due to the fact that these samples from the AR-areas were collected from surrounding industrial or commercial areas. The PBDE concentrations in vegetables in the AR-areas were generally lower than our results [46,50].

Further, the profiles of PBDEs in soils, sediments, and vegetables between this study and other areas were compared. Based on our collected literature, BDE-209 contributed a median of 84%, 45%, and 40% of total concentrations of PBDEs in the soils, sediments, and vegetables, respectively, in the IR-sites (Tables S3–S5 from Supplementary Materials). However, the penta-BDEs and octa-BDEs usually contributed a lot to total PBDE concentrations. Typically, the sum of BDE-47 and BDE-99 accounted for 27%, 63%, and 38% of the total PBDE concentrations in the soil, sediments, and vegetables in these IR-sites, respectively [36,51,52]. Historically, a large amount of e-waste mainly imported from European and American countries were irregularly recycled in China. These e-wastes usually contained penta-BDEs and octa-BDEs. In the UI-areas, BDE-209 contributed a median of 95%, 80%, and 96% of the total PBDE concentrations in soils, sediments, and vegetables, respectively, which was similar to our results [45,48,53]. In addition, the median contributions of BDE-209 ranged from 85% to 91% in the AR-areas. These results further indicated the difference in the sources of e-waste between the IR-sites and this site.

In China, the technical specifications of pollution control in e-waste recycling were developed and implemented in 2010. In the specifications, many measures including controlling dust emission, forbidding crud waste recycling, and harmless disposal of residues were required. In general, the observed PBDE concentrations in the investigated site was much lower than those in IR-sites, and comparable to those in UI-areas. Additionally, media concertation of PBDEs in sediments across China reached 15.8 ng·g^−1^, which was slightly higher than that in this study [54]. This indicated that the pollution control measures taken by this recycling plant might be effective.

### 2.2. Contamination of Heavy Metals

The descriptive statistics of the heavy metals in soils, sediments, and vegetables in this study are summarized in Table 2. With the exception of zinc, the mean concentrations of metals in this site were higher than their background values of soils [55]. Especially, the mean concentrations of Mn, Sb, and As were 2.86, 2.34, and 2.16 times as much as their background values. In general, based on China’s standard for soil risk control (GB 36600-2018 and GB 15618-2018), the concentrations of soil metals (excluding As) in the factory and in the surrounding farmland were lower than the risk screening values in industrial and agricultural areas, respectively. The concentrations of As, Cr, and Ni in this site were 1.65, 1.52, and 1.31 times as much as those in Chinese soils, while the concentrations of other metals were comparable to or lower than those across China [56]. Further, the pollutions of soil metals were assessed using the geoaccumulation index (*I*_geo_) values and the results are shown in Figure S2 from Supplementary Materials. In our study, the metals of Mn, As, Sb, Hg, and Cr ranged from unpolluted to moderately polluted, with the mean *I*_geo_ values of 0.87, 0.47, 0.45, 0.35, and 0.04, respectively. The *I*_geo_ values for Mn and Sb were 1.53 and 1.52 at the 95th percentile, belonging to moderately polluted levels. The mean *I*_geo_ values showed that the other metals in soils belonged to the unpolluted levels (*I*_geo_ ≤ 0).

The concentrations of heavy metals in sediments were higher than the soil background values (Table 2). In particular, the concentrations of Hg and Sb were 39.9 and 23.0 times as much as the corresponding background values, respectively. According to the numerical sediment quality guidelines, however, the mean concentrations of metals (excluding Cr) were between the threshold effect concentration (TEC) and probable effect concentration (PEC), indicating that the pollution might have low adverse effects [57]. In addition, the mean *I*_geo_ values showed that heavy metals in the investigated sediments were generally at unpolluted to moderately polluted levels (Figure S2). It should be noted that the maximum *I*_geo_ values of Cd, Hg, Sb, and Zn in S_24_ with 3.60, 7.44, 6.51, and 3.49, respectively, belonged to heavily and extremely polluted, likely due to the rainfall, wastewater discharge, and poor water mobility in this sampling site.

The concentrations of metals in the study vegetables differed (Table 2). The highest concentrations (ng·g^−1^) of Cd (311) and Hg (370) were observed in the pine needle and wheat samples, respectively. As for the other metals, relatively high concentrations were found in the baby’s breath samples. In particular, the Mn concentration reached 284 μg·g^−1^. This phenomenon suggested that the vegetables had species-specific absorption capacities [58].

**Figure 2:**
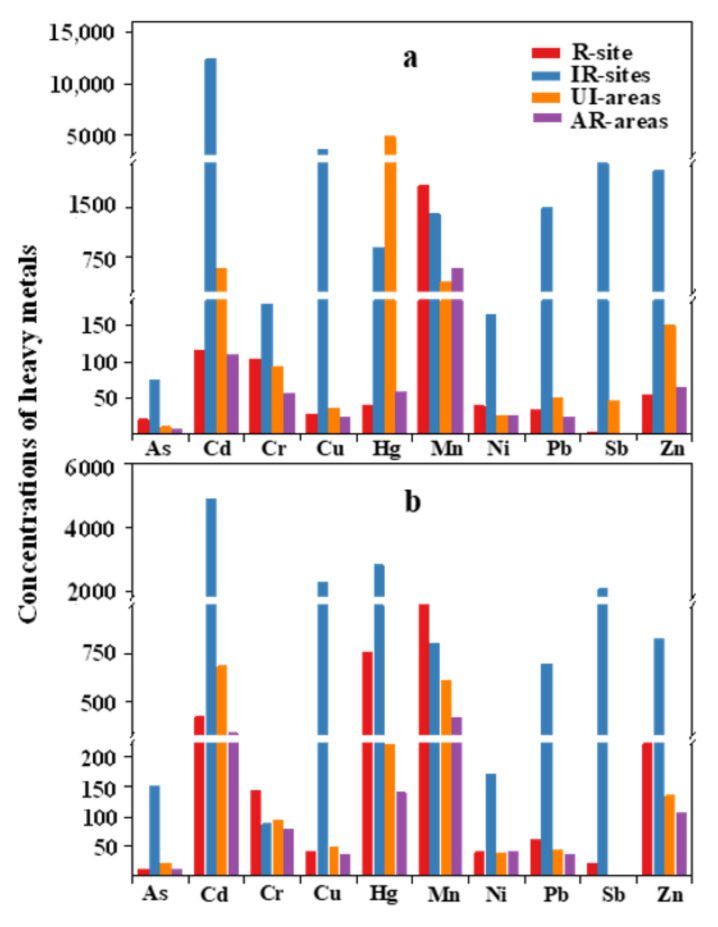
Comparison of heavy metal concentrations in soils (**a**) and sediments (**b**) in the study area with those in other areas. The units of Cd and Hg were ng·g^−1^, and those of other metals were μg·g^−1^. R-site, regulated e-waste recycling site (this study); IR-sites, irregulated e-waste recycling sites; UI-areas, urban and industrial areas; AR-areas, agricultural and rural areas. The mean concentrations of heavy metals in each study were selected. Data are from Tables S6 and S7 from Supplementary Materials.

The metal concentrations in the soils, sediments, and vegetables in this study were compared with those in some IR-sites (Tables S6–S8 from Supplementary Materials). Concentrations of Cd, Cu, Pb, Sb, and Zn in the soils in this study were 1–3 orders of magnitude lower than those from the IR-sites [28,59]. Wu et al. [60] reported mean concentrations of As and Ni in an e-waste burning site in Guiyu, which were 2.52 and 6.58 times higher than our results, respectively. However, Mn concentrations in soils found in this study were comparable to those from the e-waste recycling areas (Figure 2a). The reported concentrations of metals in the sediments were generally 1–2 orders of magnitude lower than those found in the IR-sites (Figure 2b). The Cr concentrations in the investigated sediments was 1–2 orders of magnitude higher than those in the IR-sites [61,62], suggesting that the release of heavy metals during different e-waste recycling activities differed [5]. Concentrations of As, Cu, and Zn in vegetables from this regulated site were lower than those in the IR-sites [50]. The contamination levels of Cd, Hg, Mn, Ni, and Pb were comparable to those in IR-sites [36,50,63]. Compared to the IR-sites, the concentrations of Cd, Cu, Pb, Sb, and Zn in this site were generally at lower levels, especially in soils and sediments.

The concentrations of metals found in our study were also compared to those observed in the UI-areas (Figure 2). The concentrations of heavy metals in soils in this regulated site were generally comparable to those observed in urban and industrial soils. However, the concentrations of Hg and Zn in soils in this study were 1–3 orders of magnitude lower than those from UI-areas [64,65], while concentrations of As and Ni were 2.02 to 1.52 times that in the UI-areas [64,66,67]. The concentrations of most metals, except for As and Cd, in the sediments in our study were higher than those in the UI-areas (Figure 2b). Yang et al. [68] reported that the concentrations of As and Cd in the Wuhan section of the Yangtze River were 1.23 and 1.62 times as much as those in our study, respectively. The contamination levels of Cu and Ni in the sediments were comparable to those in the UI-areas [68]. For the vegetables, the concentrations of Cd, Cu, Pb, and Zn found in this study were 1–2 orders of magnitude lower than those from UI-areas [69,70] (Table S8 from Supplementary Materials). However, Bi et al. [71] reported that the mean concentration of As in the leafy vegetables from Shanghai was two orders of magnitude lower than our study. Generally, the concentrations of metals (excluding As) in soils and vegetables in our study were comparable to or lower than those in the UI-areas, while the concentrations of sediment metals were slightly higher than those in the UI-areas.

Compared to the AR-areas, the concentrations of heavy metals in soils in this study were relatively high (Figure 2). The mean concentrations of soil heavy metals (excluding Hg and Zn) in our study were 1.06 to 3.21 times as much as those in the AR-areas (Figure 2a). In the sediments, the concentrations of all metals were generally comparable to or higher than those in the AR-areas. The concentrations of Cu, Ni, Pb, and Zn in the vegetable samples were 1–2 orders of magnitude higher than those from the AR-areas, and the contamination of Cd and Cr in the AR-areas were comparable to those in this regulated site [72,73,74]. This indicated that the regulated e-waste recycling operations could reduce the release of metals to some extent, although the metal concentrations were still higher than those in the AR-areas.

### 2.3. Health Risk Assessment

**Figure 3:**
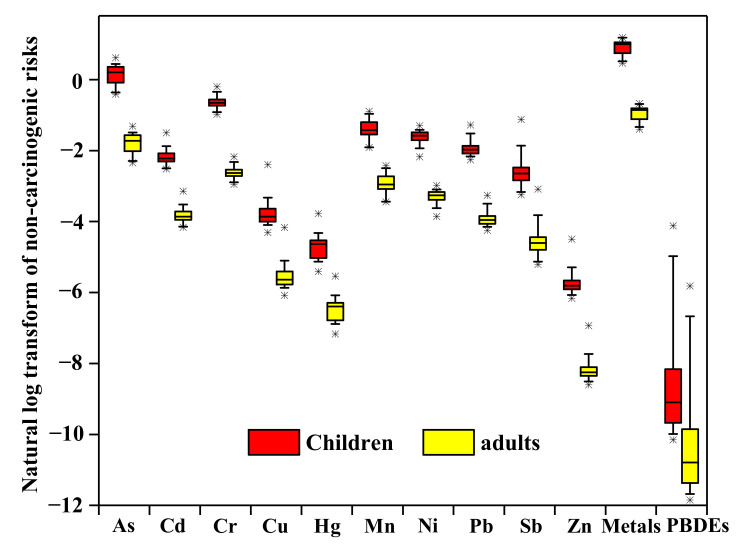
Non-carcinogenic risks to children and adults from exposure to metals and PBDEs in soils. Horizontal lines represent the 5th, 50th, and 95th percentiles, and the boxes represent the lower quartile and upper quartile; the asterisk below or above indicates outliers.

The non-carcinogenic risks from exposure to PBDEs and metals in the soils in this site were calculated to further understand the potential adverse effects on human health. Figure 3 depicts the non-carcinogenic risks for children and adults. In this study, only four congeners, i.e., BDE-47, BDE-99, BDE-153, and BDE-209, were selected to calculate the hazard quotient (HQs), due to the lack of RfDs of the other congeners. The calculated results of individual PBDEs are depicted in Table S9 from Supplementary Materials. For adults, the HIs of PBDEs in soils ranged from 7.15 × 10^−6^–2.98 × 10^−3^, and the HIs of metals ranged from 0.25–0.51, indicating that the risks from PBDEs and metals in the soils in this area were acceptable. For children, the HIs of PBDEs in soils ranged from 3.89 × 10^−5^–1.62 × 10^−2^; however, the HIs of soil metals ranged from 1.59–3.27, indicating the unacceptable non-carcinogenic risks to children. As, Cr, and Mn were the main contributors to the total risks, with a median of 47%, 21%, and 10% of the HIs. In particular, the HQs of As in 71.4% of the total soil samples were higher than 1.0, which exceeded the acceptable level.

Many studies reported that the non-carcinogenic risks from these chemicals in IR-sites were much higher than the risk levels in our study. Typically, Ge et al. [75] reported that the non-carcinogenic risks of PBDEs for children in an e-waste dismantling site in South China, at the 95th percentile, reached 0.486, which was two orders of magnitude higher than our result (0.007). Zhang et al. [76] also found that the HQs for Pb, Cd, Hg, and Cu in an abandoned e-waste recycling plant in Taizhou were higher than 10. These results suggested that the non-carcinogenic risks from the chemicals released from the regulated e-waste recycling were substantially reduced, but further control of the risks from some specific chemicals are imperative and necessary.

## 3. Materials and Methods

### 3.1. Study Area

The regulated e-waste recycling site, surrounded by farmland, is located in a small town on the outskirt of Qingdao, Eastern China, and covers an area of about 43 hectares. The area has a warm and humid climate, and annual average temperature, precipitation, and wind speed of 11.3 °C, 732 mm, and 2–3 m·s^−1^, respectively [77]. E-waste recycling activities in this park lasted for 13 years. By the end of 2015, the annual treatment capacity of e-waste in this site reached 500,000 tons. In the site, e-waste was dismantled physically with mechanization. Many pollution-control measures, including classified storing, classified dismantling, closed crushing, negative pressure in workshop, were implemented in the recycling site, based on China’s technical specifications of pollution control for this recycling. Typically, the dust containing metals and organic pollutants was removed by adsorption in the negative pressure workshop. The fragments of printed circuit board were transported to other sites for subsequent treatment. The operations carried out in the factory followed the OHSAS 18001 guidelines.

### 3.2. Sample Collection

In June 2015, soil, sediment, and vegetable samples from this regulated e-waste recycling site were collected. The sampling sites are depicted in Figure S1 from Supplementary Materials. Based on the technical guidelines on soil sampling recommended by China [78], a total of 21 composite soil samples (0–10 cm deep) were collected in the factory (S_1_–S_7_ and S_19_–S_21_) and the surrounding farmland (S_8_–S_18_). The farmland was about 300 to 2000 m from the e-waste dismantling workshop. Each soil sample consisted of four sub-samples. According to the Chinese specification [79], seven composite sediment samples (ca. 1 kg) were collected randomly from the surrounding ditches (S_22_–S_28_). Each sediment sample consisted of four sub-samples. Additionally, the wheat leaf (*Triticum aestivum* L.) (V_1_ and V_4_) was collected from the surrounding farmland. The metasequoia leaf (*Metasequoia glyptostroboides*) (V_2_), pine needle (*Pinus massoniana* Lamb) (V_3_), rattan grass (*Vulpia myuros*) (V_5_), aspen leaf (*Populus alba* L.) (V_6_ and V_8_), and a whole plant of baby’s breath (*Gypsophila paniculata* L.) (V_7_) were collected within the site. Four parallel samples were randomly selected for each vegetable. The soil and vegetable samples were wrapped in sealed aluminum foil bags (washed with acetone before being used). The sediment sample was placed in a washed aluminum box. All samples were freeze-dried and then sealed before being ground, homogenized, sieved (a 150-mesh, approximately 100-μm, stainless sieve), and stored at −20 °C, until analysis within two months.

### 3.3. Sample Analysis

In the study, twenty-one PBDE congeners and ten metals in the soil, sediment, and vegetable samples were measured. The extraction and cleanup procedures for the detection of PBDEs were conducted on the basis of the methods described by Tang et al. [80], which are described in the Section 1 in Supplementary Materials. The selected PBDE congeners were analyzed by a gas chromatography/mass spectrometry (Agilent 7890A/5975C, Agilent Technologies, Santa Clara, CA, USA), and the instrumental conditions were described in the Section 2 in Supplementary Materials. A spiked blank, procedural blank, and matrix-spiked sample was measured after every 8–10 samples, to monitor the performance of the method and matrix effects. All samples were determined twice. The recoveries of PBDEs in spiked soil/sediment and vegetable samples were 81.5−127% and 81.0−127%, and their relative standard deviations (RSDs) were 4.52−16.6% and 4.52−16.5%, respectively. The limit of quantification (LOQ) for PBDEs in the soil/sediment and vegetable samples were in the range of 0.002−0.250 and 0.003−0.252 ng·g^−1^ dry weight, respectively, based on a signal-to-noise ratio of 10:1.

For metal measure, the soil and sediment samples were digested by the HNO_3_: HF: HClO_4_ (*v*:*v*:*v* = 5:10:2) method, which was described by Tang et al. [81]. The vegetable samples were digested with a microwave digestion system, based on the methods described by Cheng et al. [58]. The sample preparation is described in the Section 1 in Supplementary Materials. The concentrations of As and Hg were analyzed using an atomic fluorescence spectrometer (XGY-1011A, IGGE, Langfang, China), and the concentrations of the other eight metals were determined with an inductively coupled plasma mass spectrometer (Agilent 7500a, Agilent Technologies, Santa Clara, CA, USA). Reagent blanks and procedural blanks were analyzed to check for interference with the analysis. The duplicate samples were analyzed to evaluate the precision of the analysis (produced values within ±10%). Geochemical standard reference soil samples (GSS-17 and GSS-25) and vegetable samples (GSB-2, GSB-4, and GSB-5) were analyzed and the relative standard deviations (RSDs) of the results were generally better than 10%. All samples were determined twice.

### 3.4. Statistical Analysis and Risk Assessment

Statistical analyses of PBDE and metal concentrations in soils, sediments, and vegetables were conducted using SPSS 18.0 (IBM SPSS, Armonk, NY, USA). Origin 8.0 software was used to draw the figures (Origin Lab, Northampton, MA, USA). For concentrations below LOQ, the value was set to half of LOQ during data analysis. The Kolmogorov–Smirnov test was used to test the normality of data. In this study, the observed concentrations of PBDEs and metals were compared with those reported in some previous investigation in irregulated e-waste recycling sites (IR-sites), urban and industrial areas (UI-areas), and agricultural and rural areas (AR-areas). For the IR-sites, only the literature reported for the mechanical recycling site was selected. The information about the pollution from the PBDE manufacturing and metal smelting was excluded in the data screening for the UI-areas. In addition, the PBDEs and metal concentrations reported for agricultural areas close to cities or remote areas were also excluded from the literature collection for AR-areas. After screening and sorting, the PBDE concentrations reported in 37 references and metal concentrations from 44 references were collected, and the information was depicted in Tables S3–S8 from Supplementary Materials, respectively.

The *I*_geo_ was used to evaluate the metal pollution in the soil and the sediment samples. The calculation method is described in Section 3 in Supplementary Materials. According to Muller et al. [82], the corresponding relationships between I_geo_ and the levels of pollution were defined as follows—unpolluted (*I*_geo_ ≤ 0), unpolluted to moderately polluted (0 < *I*_geo_ ≤ 1), moderately polluted (1 < *I*_geo_ ≤ 2), moderately to heavily polluted (2 < *I*_geo_ ≤ 3), heavily polluted (3 < *I*_geo_ ≤ 4), heavily to extremely polluted (4 < *I*_geo_ ≤ 5), and extremely polluted (*I*_geo_ ˃ 5). In addition, the non-carcinogenic risks from exposure to metals and PBDEs in soils were also estimated. According to the Exposure Factors Handbook [83], the average daily doses (ADDs) (mg·kg^−1^·day^−1^) through ingestion, dermal contact, and inhalation for both adults and children were estimated using Equations (1)–(3), as follows:

ADD_ingest_ = C_soil_ × IngR × EF × ED / BW / AT × 10^−6^ (1)

ADD_dermal_ = C_soil_ × SA × AF × ABS × EF × ED / BW / AT × 10^−6^ (2)

ADD_inhale_ = C_soil_ × InhR × EF × ED / PEF / BW / AT (3)

where *ADD*_ingest_, *ADD*_dermal_, and *ADD*_inhale_ are the daily exposure to metals through soil ingestion, dermal contact, and inhalation absorption, respectively; *C*_soil_ is the concentration of metal in soil; *IngR* and *InhR* are the ingestion and inhalation rates of soil, respectively; *EF* is the exposure frequency; *ED* is the exposure duration; *BW* is the body weight of the exposed individual; *AT* is the time period over which the dose is averaged; *SA* is the exposed skin surface area; *AF* is the adherence factor; and *PEF* is the particle emission factor. The value for each factor was selected according to the literature, which is shown in Table S1 from Supplementary Materials. *ABS* is the dermal absorption factor; given in Table S2 from Supplementary Materials. The non-carcinogenic risks from metals could be assessed using the hazard quotient (HQ), which is the ratio of the ADD to the reference dose (RfD, mg·kg^−1^·day^−1^) of a metal, for the same exposure pathway. The value of RfD for each metal is given in Table S2 from Supplementary Materials. In this study, the hazard index (HI) was defined as the sum of the hazard quotient (HQ) values. If the hazard index HI ≤ 1, the non-carcinogenic risks were acceptable. The potential non-carcinogenic health effects occurred when HI > 1 [84].

## 4. Conclusions

This study reports the concentrations of PBDEs and metals in the soils, sediments, and vegetables surrounding regulated e-waste recycling sites in China. The results indicated that the concentrations of these typical chemicals in this site were much lower than those in irregulated e-waste recycling sites, and are generally comparable to those in the urban and industrial areas. This study suggested that the regulated operations can substantially reduce the pollution of PBDEs and metals from the e-waste recycling. The non-carcinogenic risks from these chemicals were also reduced in comparison to those in the irregulated e-waste recycling areas. However, this study found that the concentrations of some specific chemicals in this site were still relatively high. In particular, arsenic pollution in the recycling site were relatively serious and the non-carcinogenic risks from soil arsenic to local children exceeded the acceptable level. This suggested that further pollution control in regulated e-waste recycling is needed.

It should be noted that this is a pilot study with a small set of samples, and some limitations should be noted. The reported concentrations of chemicals depended on many factors, including the types of e-waste, the content of chemicals in the waste, the amount of recycling waste, the method of recycling, and the time of plant operation, as well as the local climate conditions. There might also have been some uncertainty associated with the selection of other studies, for comparison in this study. For example, the sample size and sampling times in each study varied. The difference of plant species and the growth period of plants might cause uncertainty in the evaluation of vegetable pollution in this site. In addition, the nature of the risk assessment also renders the findings uncertain. In particular, the bioavailability of the chemicals in soils were not considered in this study, which likely resulted in an overestimation of the potential risks. More importantly, the occurrence and the associated risks from many other hazardous chemicals released from the e-waste recycling were not investigated. More research is need to investigate the risks from multiclass pollutants surrounding the site and the effectiveness of the current pollution control in the e-waste recycling operation.

## Figures and Tables

**Table 1:** Summary of polybrominated diphenyl ether (PBDE) concentrations (ng·g^−1^ dry weight), with basic statistical parameters, for the soils, sediments, and vegetables from the regulated electronic waste recycling site.

	Soils		Sediments		Vegetables	
	Min–Max	Median	Min–Max	Median	Min–Max	Median
BDE-28	<0.002	<0.002	<0.002–0.043	<0.002	<0.003–0.18	0.05
BDE-47	<0.003–0.11	<0.003	<0.003–0.81	<0.003	0.005–1.27	0.26
BDE-66	<0.006–0.03	<0.006	<0.006–0.62	<0.006	<0.007–0.19	0.05
BDE-85	<0.013	<0.013	<0.013	<0.013	<0.013–0.18	<0.013
BDE-99	<0.007–0.22	<0.007	<0.007–0.31	<0.007	<0.007–0.30	0.110
BDE-100	<0.010	<0.010	<0.010–1.26	<0.010	<0.010–0.36	0.02
BDE-138	<0.005	<0.005	<0.005	<0.005	<0.005–0.06	<0.005
BDE-153	<0.008–0.33	<0.008	<0.008–0.23	<0.008	<0.008–0.20	<0.008
BDE-154	<0.022–14.0	<0.022	<0.022–0.93	0.12	<0.022–0.76	0.20
BDE-183	<0.018–0.81	<0.018	<0.018–0.13	<0.018	<0.019–0.30	0.019
BDE-190	<0.035	<0.035	<0.035	<0.035	<0.035	<0.035
BDE-196	<0.054–1.09	<0.054	<0.054	<0.054	<0.054–0.44	<0.054
BDE-197	<0.073–1.04	<0.073	<0.073–0.70	<0.073	<0.073–0.37	<0.073
BDE-201	<0.002–0.74	<0.002	<0.002–0.73	<0.002	<0.003–0.46	<0.003
BDE-202	<0.002–0.55	<0.002	<0.002–0.54	<0.002	<0.003–0.37	<0.003
BDE-203	<0.105–1.56	<0.105	<0.105	<0.105	<0.105–0.37	<0.105
BDE-205	<0.079	<0.079	<0.079	<0.079	<0.079	<0.079
BDE-206	<0.045–8.70	<0.045	<0.045–1.74	<0.045	0.34–1.76	0.89
BDE-207	<0.081–3.96	<0.081	<0.081–4.12	<0.081	<0.082–1.12	0.61
BDE-208	<0.183–1.99	<0.183	<0.183–1.81	<0.183	<0.184–1.24	0.54
BDE-209	3.05–1,331	8.09	4.27–314	10.8	90.2–419	201
Σ_21_PBDEs	3.05–1,336	9.23	4.31–327	11.6	93.9–425	204

**Table 2:** Descriptive statistics of metal concentrations (μg·g^−1^) in soils, sediments, and vegetables collected from a regulated electronic waste recycling site in China.

Sampling Sites	Statistics	As	Cd	Cr	Cu	Hg	Mn	Ni	Pb	Sb	Zn
Soils (*n* = 21)	Range	11.2–31.3	0.08–0.22	73.2–159	14.9–101	0.02–0.09	1077–2991	21.8–52.4	23.7–63.3	0.96–8.07	32.2–170
	Mean	20.0	0.12	104	27.9	0.04	1839	38.9	33.4	2.11	54.0
Background values ^1^		9.30	0.08	66.0	24.0	0.019	644	25.8	25.8	0.90	63.5
Risk screening values		60 ^2^	65 ^2^		18,000 ^2^	38 ^2^	–	900 ^2^	800 ^2^	–	–
		25 ^3^	0.6 ^3^	250 ^3^	100 ^3^	3.4 ^3^	–	190 ^3^	170 ^3^	–	300 ^3^
Sediments (*n* = 7)	Range	7.41–18.0	0.11–1.53	98.1–294	17.5–132	0.03–4.95	780–1684	27.3–61.8	26.3–226	1.52–123	52.9–1071
	Mean	12.0	0.42	144	41.6	0.76	1145	40.7	62.3	20.7	226
TEC ^4^		9.79	0.99	43.4	31.6	0.18		22.7	35.8		121
PEC ^4^		33.0	4.98	111	149	1.06		48.6	128		459
Vegetables (*n* = 8)	Range	0.49–1.72	0.03–0.31	3.28–13.4	4.00–16.5	0.05–0.37	86.7–284	1.30–6.14	1.09–9.93	0.06–2.76	21.8–73.7
	Mean	0.95	0.14	5.49	9.99	0.17	177	3.39	3.94	0.62	42.2

^1^ Background values (μg·g^−1^) of metals in soils in Shandong Province, China [55]. ^2^ Limits in the Chinese standard (GB 36600–2018). ^3^ Limits in the Chinese standard (GB 15618–2018). ^4^ TEC, the threshold effect concentration; PEC, the probable effect concentration [57].

## Data Availability

Not applicable.

## Supplementary Materials

The following are available online, Sample preparation, Instrumental Analysis, Geoaccumulation index, Table S1: Exposure factors and values used in the non-carcinogenic risk estimation, Table S2: Reference dose of different exposure routes and skin absorption factors, Table S3: Polybrominated diphenyl ether concentrations in soils from the regulated e-waste recycling area and from other areas, Table S4: Polybrominated diphenyl ether concentrations in sediments from the regulated e-waste recycling area and from other areas, Table S5: Polybrominated diphenyl ether concentrations in vegetables from the regulated e-waste recycling area and from other areas, Table S6, Mean metal concentrations in soils from the regulated e-waste recycling area and from other areas, Table S7: Mean metal concentrations in sediments from the regulated e-waste recycling area and from other areas, Table S8: Mean metal concentrations in vegetables from the regulated e-waste recycling area and from other area, Table S9: Non-carcinogenic risks for individual polybrominated diphenyl ethers in soils, Figure S1: Map of the soil, sediment, vegetable sampling sites, Figure S2: Box plots of the geoaccumulation index for heavy metals in soils and sediments. References [28,36,37,38,39,40,41,42,43,44,45,46,47,48,49,50,51,52,53,56,58,59,60,61,62,63,64,65,66,67,68,69,70,71,72,73,74,80,81,82,84,85,86,87,88,89,90,91,92,93,94,95,96,97,98,99,100,101,102,103,104,105,106,107,108,109,110,111,112,113,114,115,116,117,118,119,120,121,122,123,124,125,126,127,128,129,130,131,132,133,134,135] are cited in the Supplementary Materials.

## Sample Availability

Samples of soils, sediments, and vegetables are not available from the authors.
