# Supplementary material for: Polybrominated Diphenyl Ethers and Heavy Metals in a Regulated E-Waste Recycling Site, Eastern China: Implications for Risk Management
Source: Molecules. 2021 Apr 9;26(8):2169. doi: 10.3390/molecules26082169 (PMC8069465; doi:10.3390/molecules26082169)
Supplement: Supplementary file 1 [file molecules-26-02169-s001.pdf]

molecules

Supplementary Material

Polybrominated Diphenyl Ethers and Heavy Metals in a Regulated E-Waste Recycling Site, Eastern China: Implications for Risk Management

Hongmin Yin [1], Jiayi Ma [2], Zhidong Li [3], Yonghong Li [2], Tong Meng [1] and Zhenwu Tang [1,2,*]

[1] College of Environmental Science and Engineering, North China Electric Power University, Beijing 102206, China; hongminyin@ncepu.edu.cn (H.Y.); mengtong@ncepu.edu.cn (T.M.)
[2] College of Life and Environmental Sciences, Minzu University of China, Beijing 100081, China; 17010176@muc.edu.cn (J.M.); 20400254@muc.edu.cn (Y.L.)
[3] Cangzhou Ecology and Environment Bureau, Cangzhou 061000 Hebei, China; lzdhdm@163.com
* Correspondence: zwtang@muc.edu.cn; Tel.: +86–10–68936927

This file has 14 pages and includes two figures and nine tables.

Section 1

Sample Preparation

The polybrominated diphenyl ethers (PBDEs) in soils, sediments and vegetables were determined used the method described by Tang et al. [1], with some modifications. Each sample (10.0 g) was extracted using a 1:1 (*v*/*v*) mixture of *n*-hexane and dichloromethane in an ultrasonic bath for 30 min, twice. The extracts were combined and desulfurized by copper. The concentrated extracts were passed through a 10 mm inner diameter glass column containing neutral alumina (6 cm), neutral silica gel (2 cm), alkaline silica gel (5 cm), neutral silica gel (2 cm), acidic silica gel (8 cm) and anhydrous sodium sulfate (2 cm), and then eluted with a mixture of 70 mL hexane and dichloromethane (*v*/*v*, 1:1). The extraction solution was concentrated to 0.2 mL, and amount of ^{13}C-PCB-208 was added as internal standard, and analyzed by a gas chromatography/mass spectrometry (GC/MS).

Heavy metals in soils and sediment samples were measured with the method described by Tang et al. [2]. Briefly, 0.25 g of sample was added to the mixed solution (5.0 mL HNO_3 + 10 mL HF + 2.0 mL $HClO_4$), and then heated to 200 °C until the liquid had evaporated. 8 mL of aqua regia were added and the mixture was heated. When the volume is reduced to 2–3 mL, the digestive solution was diluted to 25 mL with deionized water. Then 1 mL of the solution was selected and diluted to 10 mL with 2% nitric acid for analysis. Vegetable samples were digested by a microwave method described by Cheng et al. [3]. A total of 0.50 g of vegetable sample was put into a Teflon vessel and then the mixed solution (5.0 mL HNO_3 + 2.0 mL H_2O_2) was added, and then digested with the following conditions: 2 min for 250 W, 2 min for 0 W, 6 min for 250 W, 5 min for 400 W, 8 min for 550 W, vent: 8 min. After digestion and cooling, the solution was filtered and diluted to 10 mL with 1 mol·L^{-1} HNO_3 for analysis.

Section 2

Instrumental Analysis

The quantitative analysis of the PBDEs was carried out using an Agilent 7890 gas chromatograph coupled to an Agilent 5975 mass spectrometer (Agilent 7890A/5975C, Agilent Technologies, Santa Clara, CA, USA) using a negative chemical ionization source in selected ion monitoring mode. A DB-5MS column (15 m × 0.25 mm × 0.1 µm; Agilent) was used for separation of PBDE homologues. The temperature of ion source was 150 °C. The

GC oven temperature program was 80 °C for 0.75 min, increased to 240 °C at 10 °C·min^{-1}, then at 20 °C·min^{-1} increased to 290 °C and held for 10 min. The injector temperature was 250 °C, and the helium mobile phase flow rate was 1.0 mL·min^{-1}. The injector was used in the splitless mode for the less brominated PBDEs (BDE-28, BDE-47, BDE-66, BDE-85, BDE-99, BDE-100, BDE-138, BDE-153, BDE-154, BDE-183, and BDE-190) and the split mode for more heavily brominated PBDEs (BDE-196, BDE-197, BDE-201, BDE-202, BDE-203, BDE-205, BDE-206, BDE-207, BDE-208, and BDE-209).

The concentrations of Cr, Cu, Zn, Cd, Sb, and Pb were determined using an inductively coupled plasma mass spectrometer (Agilent 7500a, Agilent Technologies, Santa Clara, CA, USA), while concentrations of As and Hg were measured using an atomic fluorescence spectrometer (XGY-1011A, IGGE, Langfang, China).

Section 3

Geoaccumulation Index (I_{geo})

The I_{geo}, introduced by Muller [4], has been used widely used in heavy metal pollution research. The equation $I_{geo} = \log_2 (C_n/1.5B_n)$ was used to calculate the geoaccumulation index, where C_n is the measured concentration of the metal in soil or sediment, and B_n is the geochemical background concentration of the metal. We selected the background values for soil metal in Shandong, China, as the B_n values, which were shown in Table 2 (see article). The coefficient 1.5 was used to amplify very small anthropogenic influences.

Table S1. Exposure factors and values used in the non-carcinogenic risk estimation.

Parameter	Definition	Unit	Value		Reference
			Children	Adult	
IngR	Soil ingestion rate	mg/day	200	100	[5]
InhR	Inhalation rate	m^3/day	5	15	[6]
EF	Exposure frequency	day/year	345	345	[7]
ED	Exposure duration	year	24	6	[5]
BW	Average body weight	kg	15	60	[6]
AT	Average time	day	365 × ED	365 × ED	[8]
SA	Exposed skin surface area	m^2	1600	4350	[6]
AF	Skin adherence factor	mg/(cm^2 day)	0.7	0.2	[9]
PEF	Particle emission factor	m^3/kg	1.36×10^9	1.36×10^9	[5]

Table S2. Reference dose (RfDs) of different exposure routes and skin absorption factors (ABS) for heavy metals and PBDEs.

Compound	Ingestion	Inhalation	Dermal contact	ABS
As	0.0003 [1]	0.0003 [1]	0.000123 [1]	0.03 [2]
Cd	0.001 [3]	0.001 [3]	0.00001 [3]	0.14 [2]
Cr	0.003 [1]	0.000029 [1]	0.003 [1]	0.04 [2]
Cu	0.04 [1]	0.042 [1]	0.012 [1]	0.1 [2]
Hg	0.00016 [3]	0.0000857 [3]	0.000021 [3]	0.05 [2]
Mn	0.14 [1]	0.000014 [1]	0.023 [1]	0.01 [3]
Ni	0.02 [1]	0.021 [1]	0.0054 [1]	0.35 [2]
Pb	0.0035 [1]	0.00352 [1]	0.000525 [1]	0.006 [2]
Sb	0.0004 [3]	0.000014 [3]	0.000008 [3]	0.001 [3]
Zn	0.3 [1]	0.3 [1]	0.06 [1]	0.02 [2]

BDE-47	0.0001 [4]	0.0001 [8]	0.0001 [8]	1 [8]
BDE-99	0.0001 [5]	0.0001 [8]	0.0001 [8]	1 [8]
BDE-153	0.0002 [6]	0.0002 [8]	0.0002 [8]	1 [8]
BDE-209	0.007 [7]	0.007 [8]	0.007 [8]	1 [8]

[1] [10]; [2] [11]; [3] [6]; [4] [12]; [5] [13]; [6] [14]; [7] [15]; [8] In this study, the inhalation RfD and dermal RfD of BDE-47, 99, 153 and BDE-209 are assumed to be equal to its corresponding ingestion RfD, respectively; the values of ABS were all assumed to be equal to 1.

Table S3. Polybrominated diphenyl ether (PBDE) concentrations (ng·g^{-1} dry weight) in soils from the regulated e-waste recycling area and from other areas.

Locations	Sampling Times	Types	BDE-209 (max–min) Median/Mean	∑PBDEs (max–min) Median/Mean	References
**Qingdao, China**	**2015**	**Regulated e-waste recycling site**	**(3.05–1,331) 8.09**	**(3.05–1,366) 9.23**	**This study**
Guiyu, China	2009–2010	Irregulated e-waste recycling sites	(ND–12,130) 10,090	(2.00–71,840) 44,360	[16]
Guiyu, China	2004	Irregulated e-waste recycling sites	(510–1,270) 890	(1,440–3,570) 2,505	[17]
Guiyu, China	2014	Irregulated e-waste recycling sites	14,107	17,930	[18]
Qingyuan, China	-	Irregulated e-waste recycling sites	(21.7–23,131) 5,568	(28.2–23,517) 5,813	[19]
Qingyuan, China	-	Irregulated e-waste recycling sites	(4.40–21,467) 2,126	(5.27–22,108) 2,283	[19]
Longtang, China	2012	Irregulated e-waste recycling sites	(347–3,340) 1,994	(421–3,713) 2,273	[20]
Taizhou, China	2007	Irregulated e-waste recycling sites	(69.9–5,530) 1,800	(71.6–5,710) 1,910	[21]
Zhejiang, China	2008	Irregulated e-waste recycling sites	(1.20–356) 148	654	[22]
Shanghai, China	2007	Urban and industrial areas	(1.93–268) 40.6	(2.03–269) 40.6	[21]
Tianjin, China	2018	Urban and industrial areas	(0.93–106) 9.16	(1.11–108) 9.76	[23]
Shanghai, China	2006	Urban and industrial areas	(0.001–2.91) 0.48	(0.024–3.80) 0.74	[24]
China	2012	Urban and industrial areas	(0.47–790) 72.0	(0.60–800) 75.0	[25]
Taiyuan, China	2006	Urban and industrial areas	(0.006–210) 13.3	(0.016–211) 14.7	[26]
Shen-Fu, China	2016	Urban and industrial areas	(ND–39.1)	(0.28–50.7) 10.5	[27]
Shanghai, China	2012	Urban and industrial areas	(4.74–35.6) 18.0	(5.19–40.5) 19.7	[28]
Baotou, China	2017	Urban and industrial areas	(1.59–64.3) 13.1	13.3	[29]
Huhhot, China	2017	Urban and industrial areas	(2.13–47.9) 5.91	5.99	[29]
Shanghai, China	2007	Agricultural and rural areas	(0.03–0.80) 0.25	(0.13–1.25) 0.43	[30]

The Yangtze River Delta, China	2014	Agricultural and rural areas	(ND–109) 3.01	(ND–382) 4.02	[31]
Shanghai, China	2012	Agricultural and rural areas	(3.55–48.7) 16.1	(4.31–49.3) 17.7	[28]
Baynnur, China	2017	Agricultural and rural areas	(0.51–3.97) 1.4	1.45	[29]
Tongliao, China	2017	Agricultural and rural areas	(0.69–2.25) 1.31	1.36	[29]

Table S4. Polybrominated diphenyl ether (PBDE) concentrations (ng·g^{-1} dry weight) in sediments from the regulated e-waste recycling area and from other areas.

Location	Sampling Times	Types	BDE-209 (max–min) Median/Mean	∑PBDEs (max–min) Median/Mean	References
**The ditches in Qingdao, China**	**2015**	**Regulated e-waste recycling site**	**(4.27–314) 10.8**	**(4.31–328) 11.6**	**This study**
The river in Longtang, China	2012	Irregulated e-waste recycling sites	(384–4,449) 2,405	(483–5,314) 5,314	[20]
A pond in Qingyuan, China	2016	Irregulated e-waste recycling sites	-	(65–98,000) 530	[32]
A pond in Qingyuan, China	2016	Irregulated e-waste recycling sites	-	(122–125,000) 510	[32]
A pond in Qingyuan, China	2016	Irregulated e-waste recycling sites	-	(5,000–1,030,000) 43,000	[32]
The rivers in Guiyu, China	2006	Irregulated e-waste recycling sites	(7.02–66,573) 8,840	(9.59–87,779) 9,054	[33]
The Nanyang River, China	2004	Irregulated e-waste recycling sites	(16.9–62.2) 35.9	(4,434–16,088) 9,357	[34]
The Yongning River, China	2014	Irregulated e-waste recycling sites	(300–25,751) 10,108	(303–26,838) 10,363	[35]
The river in South China, China	2013	Irregulated e-waste recycling sites	(740–1,000) 910	(2,000–4,700) 2,900	[36]
The Pearl River, China	2006	Urban and industrial areas	(12.2–488) 123	(12.5–524) 127	[33]
The Zhujiang River, China	2002	Urban and industrial areas	(26.3–3,575) 890	(27.7–3,587) 903	[37]
The Yangtze River Delta, China	2012	Urban and industrial areas	56.6–2,059	(57.1–2,061) 305	[38]
Lake Chaohu, China	2010	Urban and industrial areas	(2.44–72.5) 20.5	(3.64–86.7) 25.5	[39]
Lake Taihu, China	2016	Urban and industrial areas	(16.6–160) 46.8	(20.1–180) 53	[40]
Maanshan, China	2018	Urban and industrial areas	(0.02–26.6) 6.00	(0.02–106) 30.7	[41]
Wuhu, China	2018	Urban and industrial areas	(0.01–19.4) 5.35	(0.01–77.6) 23.1	[41]
Tongling, China	2018	Urban and industrial areas	(0.54–35.8) 6.04	(0.01–84.8) 20.5	[41]
The Haihe River, China	2017	Agricultural and rural areas	(1.10–4.90) 2.40	(0.27–4.9) 3.40	[42]

The Xijiang River, China	2002	Agricultural and rural areas	(1.90–77.4) 5.68	(2.10–77.9) 6.04	[37]
Lake Chaohu, China	2010	Agricultural and rural areas	(2.44–72.5) 20.5	(3.64–86.7) 25.5	[39]
The Maozhou River, China	2010	Agricultural and rural areas	(1.93–101) 16.9	(2.11–128) 19.0	[43]

Table S5. Polybrominated diphenyl ether (PBDE) concentrations (ng·g^{-1} dry weight) in vegetables from the regulated e-waste recycling area and from other areas.

Location	Sampling Times	Types [1]	BDE-209 (max–min) Median/Mean	∑PBDEs (max–min) Median/Mean	References
**Qingdao, China**	**2015**	**Wheat leaf**	**(94.9–204) 185**	**(100–205) 188**	**This study**
**Qingdao, China**	**2015**	**Metasequoia leaf**	**(246–322) 284**	**(249–327) 288**	**This study**
**Qingdao, China**	**2015**	**Pine needle**	**(133–138) 136**	**(134–139) 137**	**This study**
**Qingdao, China**	**2015**	**Rattan grass**	**(198–268) 232**	**(202–272) 237**	**This study**
**Qingdao, China**	**2015**	**Aspen leaf**	**(90.2–378) 181**	**(93.9–383) 183**	**This study**
**Qingdao, China**	**2015**	**Baby's breath**	**(295–419) 357**	**(300–425) 362**	**This study**
Taizhou, China	2007	Tree and shrub leaf (IR-sites)	(1.45–40.3) 12.3	(17.1–64.2) 30.6	[21]
Taizhou, China	2006	Tree bark (IR-sites)	19.5	25.3	[44]
Taizhou, China	2008	*C. Camphora* leaf (IR-sites))	(ND–2.32)	(0.46–400)	[45]
Southern China	2007–2009	Eucalyptus leaf (IR-sites)	(6.87–45.2) 25.6	(18.9–103) 65.5	[46]
Southern China	2007–2009	Pine needle (IR-sites)	(15.6–75.1) 40.5	(27.9–383) 145	[46]
South China	2006	*Erigeron annuus* (L.) leaf (IR-sites))	15	326	[47]
Longtang, China	2012	Herb plants (IR-sites)	(3.32–76.3) 39.1	(4.84–124) 52.1	[20]
Guiyu, China	2009–2010	Edible plant (IR-sites)	(0.24–0.86) 0.55	(0.92–1.39) 1.16	[16]
Guiyu, China		Pumpkin root (IR-sites))	35.1	132	[48]
Guiyu, China	2012	Vegetable shoots (IR-sites)	(1.75–143) 17.8	(10.3–164) 37.5	[49]
Liushi, China	2009–2010	Grass leaf (UI-areas)	-	(22–223) 75.1	[50]
Ningbo, China	2009–2010	Grass leaf (UI-areas)	-	(1.40–25.6) 4.00	[50]
Shanghai, China	2015	Pine needle (UI-areas)	(Nd–324) 1.69	(3.71–4,020) 237	[51]
Baotou, China	2017	Willow bark (UI-areas)	(1.35–1.75) 1.60	1.64	[29]
Huhhot, China	2017	Willow bark (UI-areas)	(0.50–5.96) 1.67	1.73	[29]
Suzhou, China	2009	Camphor bark (UI-areas)	(82–7,420) 927	(112–7,460) 950	[52]
Nantong, China	2009	Camphor bark (UI-areas)	(148–3,240) 950	(182–3,290)1,059	[52]
Wuxi, China	2009	Camphor bark (UI-areas)	(121–2,590) 542	(156–2,600) 563	[52]

Longtang, China	2012	Herb plants (AR-areas)	(15.0–18.2) 16.3	(19.0–23.3) 21.7	[20]
Baynnur, China	2017	Willow bark (AR-areas)	(1.09–1.17) 1.13	1.21	[29]
Tongliao, China	2017	Willow bark (AR-areas)	(0.17–1.04) 0.42	0.49	[29]

[1] IR-sites, irregulated e-waste recycling sites; UI-areas, urban and industrial areas; AR-areas, agricultural and rural areas.

Table S6. Mean metal concentrations (μg·g^{-1}) in soils from the regulated e-waste recycling area and from other areas.

Location	Types	As	Cd	Cr	Cu	Hg	Mn	Ni	Pb	Sb	Zn	References
**Qingdao, China**	**Regulated e-waste recycling site**	**20.0**	**0.12**	**104**	**27.9**	**0.04**	**1,839**	**38.9**	**33.4**	**2.11**	**54.0**	**This study**
Guiyu, China	Irregulated e-waste recycling sites	-	1.96	154	788	-	3,374	114	1,431	-	-	[16]
Guiyu, China	Irregulated e-waste recycling sites	-	6.82	318	1,568	-	410	362	1,423	500	2,978	[18]
Guiyu, China	Irregulated e-waste recycling sites	70.4	5.30	277	1,207	-	420	295	1,001	3,834	-	[53]
Longtang, China	Irregulated e-waste recycling sites	-	17.1	-	11,140	-	-	-	4,500	-	3,690	[54]
Longtang, China	Irregulated e-waste recycling sites	-	10.3	63.3	4,851	-	-	100	1,715	-	1,017	[55]
Longtang, China	Irregulated e-waste recycling sites	-	39.3	-	6,372	-	-	-	1,635	-	3,040	[56]
Longtang, China	Irregulated e-waste recycling sites	80.2	6.33	-	2,159	1.38	-	78.1	576	-	1,366	[20]
Shijiao, China	Irregulated e-waste recycling sites	-	21.3	-	4,001	-	-	-	944	-	2,045	[56]
Taizhou, China	Irregulated e-waste recycling sites	-	2.60	88.6	158	0.40	-	42.9	164	-	300	[57]
Yulin, China	Urban and industrial areas	5.35	-	45.1	15	-	374	17.9	21.5	-	57.7	[10]
Guizhou, China	Urban and industrial areas	14.2	0.78	353	41.5	14.2	-	33.6	59.3	46.7	249	[58]
Liaoning, China	Urban and industrial areas	-	0.86	69.9	52.3	-	-	33.5	45.1	-	213	[59]
Guangdong, China	Urban and industrial areas	7.89	0.06	22.5	11.1	0.08	-	12	42.4	-	56.6	[60]
Beijing, China	Urban and industrial areas	-	0.15	35.6	23.7	-	-	27.8	28.6	-	65.6	[61]
Hangzhou, China	Urban and industrial areas	-	1.30	47.5	41.0	-	-	24.1	75.7	-	148	[62]
Shanghai, China	Urban and industrial areas	-	0.52	108	59.3	-	-	31.1	70.7	-	301	[63]
China, China	Urban and industrial areas	12.2	0.39	68.5	40.4	0.31	-	24.9	55.2	-	109	[64]
Heilongjiang, China	Agricultural and rural areas	8.69	0.10	53.6	18.6	0.03	-	23.3	22	-	56	[65]
Heibei, China	Agricultural and rural areas	6.16	0.15	57.8	21.2	0.08	-	25.0	18.8	-	70.0	[66]

Henan, China	Agricultural and rural areas	3.16	0.06	57.8	22.3	0.05	-	-	15.2	-	48.5	[67]
Hainan, China	Agricultural and rural areas	2.67	0.09	52.9	-	0.06	-	-	20.2	-	-	[68]
Jiangsu, China	Agricultural and rural areas	-	0.04	55	23.9	0.07	-	29.2	27.4	-	65.1	[69]
Jiangsu, China	Agricultural and rural areas	9.26	0.04	61.6	21.2	0.08	-	-	24.2	-	-	[70]
Jilin, China	Agricultural and rural areas	7.20	0.07	35	16.7	0.01	-	15.2	18.3	-	35	[71]
China	Agricultural and rural areas	-	0.21	61.4	40.5	-	573	28.2	33	1.23	92.6	[72]
China	Agricultural and rural areas	12.1	0.23	68.5	27.1	0.09	-	29.6	31.2	-	79	[73]

Table S7. Mean metal concentrations (μg·g^{-1}) in sediments from the regulated e-waste recycling area and from other areas.

Location	Types	As	Cd	Cr	Cu	Hg	Mn	Ni	Pb	Sb	Zn	References
**The ditches in Qingdao, China**	**Regulated e-waste recycling site**	**12.0**	**0.42**	**144**	**41.6**	**0.76**	**1,145**	**40.7**	**62.3**	**20.7**	**223**	**This study**
The Lianjiang River, China	Irregulated e-waste recycling sites	-	0.24	35.3	66.7	-	-	51.5	55.0	-	134	[74]
The Nanyang River, China	Irregulated e-waste recycling sites	-	6.28	65.4	2,154	-	-	294	395	-	483	[74]
The rivers in Guiyu China	Irregulated e-waste recycling sites	-	5.66	10.2	3,370	-	1,000	216	2,170	3,990	1,190	[75]
The river in Longtang, China	Irregulated e-waste recycling sites	290	11.1	-	3,393	4.08	-	134	1,170	-	2,183	[20]
A pool and dam in Qingyuan, China	Irregulated e-waste recycling sites	-	1.12	1.89	766	-	-	-	130	-	181	[76]
The Nanguan River, China	Irregulated e-waste recycling sites	12.0	6.31	317	4,788	1.55	-	153	377	-	-	[77]
The Lian River, China	Irregulated e-waste recycling sites	-	3.48	98.8	1,270	-	599	175	570	154	772	[78]
The Daliao River, China	Urban and industrial areas	-	-	-	-	-	624	-	-	0.68	-	[79]
The Yangtze River, China	Urban and industrial areas	14.2	1.10	84	50	0.14	-	39	42	-	133	[80]
The Yangtze River, China	Urban and industrial areas	14.8	0.55	113	49	0.12	-	46	43	-	165	[80]
The Yangtze River, China	Urban and industrial areas	15.2	0.40	91	41	0.14	-	42	44	-	121	[80]
The Yellow River, China	Urban and industrial areas	-	-	77.9	23	-	596	28.5	-	-	50.2	[81]
Lake Chaohu, China	Urban and industrial areas	44.4	-	102	79.4	0.49	-	-	49.5	-	206	[39]
The Yangtze River Estuary, China	Agricultural and rural areas	11.0	0.42	43.5	30.7	-	-	33.4	35.8	1.61	117	[82]
Lake Taihu, China	Agricultural and rural areas	-	0.15	95.9	40.7	-	-	49.2	52	-	95.2	[83]

The Yellow River, China	Agricultural and rural areas	11.2	0.46	79.4	32.0	0.14	-	36.1	32.1	-	118	[84]
The lakes in Wuhan, China	Agricultural and rural areas	10.0	0.32	95.5	37.4	-	420	43.7	29.5	-	96.0	[85]

Table S8. Mean metal concentrations (μg·g^{-1}) in vegetables from the regulated e-waste recycling area and from other areas.

Location	Types [1]	As	Cd	Cr	Cu	Hg	Mn	Ni	Pb	Sb	Zn	References
**Qingdao, China**	**Regulated e-waste recycling site**	**0.95**	**0.14**	**5.49**	**9.99**	**0.17**	**177**	**3.39**	**3.94**	**0.62**	**42.2**	**This study**
Guiyu, China	Edible plant samples (IR-sites)	-	2.92	2.66	29.0	-	123	13.4	11.4	-	-	[16]
Longtang, China	Vegetables (IR-sites)	-	2.62	-	20.4	-	-	-	5.79	-	135	[54]
Longtang, China	Rice (IR-sites)	-	0.43	-	42.3	-	-	-	13.6	-	94	[54]
Longtang, China	Wild plants (IR-sites)	-	1.59	-	94.5	-	-	-	54	-	143	[54]
South China	Rice (IR-sites)	-	0.44	-	20.3	-	-	1.33	0.20	-	33.1	[86]
Longtang, China	Herb plants (IR-sites)	8.63	0.34	-	96.1	0.17	-	ND	3.03	-	138	[20]
Longtang, China	Tree and herb root	-	0.81	-	303	-	-	-	31.1	-	41.5	[55]
Longtang, China	Tree and herb stalk	-	0.64	-	21.8	-	-	-	7.91	-	31.8	[55]
Longtang, China	Tree and herb leaf	-	0.58	-	49.3	-	-	-	12.3	-	40.3	[55]
Shanghai, China	*Osmanthus fragran* (UI-areas)	-	0.10	-	3.90	-	-	-	1.16	-	28.8	[87]
Shanghai, China	*Metasequoia glyptostroboides* (UI-areas)	-	0.04	-	5.88	-	-	-	1.22	-	22.4	[87]
Shanghai, China	*Pittosporum tobira* (UI-areas)	-	0.11	-	3.79	-	-	-	0.85	-	38.6	[87]
Shanghai, China	Leafy vegetables (UI-areas)	0.04	0.03	-	0.04	0.67	-	-	-	-	3.96	[88]
Hefei, China	*M. grandfora* branch (UI-areas)	-	0.05	-	-	-	-	-	1.01	-	-	[89]
Zhejiang, China	Rice (AR-areas)	-	0.09	-	2.98	-	-	0.35	-	-	22.4	[90]
Dongguan, China	Vegetable (AR-areas)	-	0.13	-	1.94	-	-	-	1.72	-	14.1	[91]
Shaoguan, China	Vegetable (AR-areas)	-	0.35	-	1.36	-	-	-	1.62	-	15.1	[91]
Guangzhou, China	Vegetable (AR-areas)	-	0.07	-	1.09	-	-	-	1.21	-	7.02	[91]
The Yangtze River, China	Lotus leaf (AR-areas)	0.20	0.28	4.66	4.62	-	-	-	0.62	-	24.1	[92]

[1] IR-sites, irregulated e-waste recycling sites; UI-areas, urban and industrial areas; AR-areas, agricultural and rural areas.

Table S9. Non-carcinogenic risks for individual polybrominated diphenyl ethers (PBDEs) in soils from the regulated electronic waste recycling site.

Compound	Children			Adults		
	P_5	P_{50}	P_{95}	P_5	P_{50}	P_{95}
BDE-47	4.82×10^{-7}	4.82×10^{-7}	4.54×10^{-5}	8.86×10^{-8}	8.86×10^{-8}	8.34×10^{-6}
BDE-99	1.34×10^{-6}	1.34×10^{-6}	1.33×10^{-5}	2.46×10^{-7}	2.46×10^{-7}	2.44×10^{-6}
BDE-153	8.65×10^{-7}	8.65×10^{-7}	2.38×10^{-5}	1.59×10^{-7}	1.59×10^{-7}	4.37×10^{-6}
BDE-209	4.34×10^{-5}	9.61×10^{-5}	6.87×10^{-3}	7.97×10^{-6}	1.77×10^{-5}	1.26×10^{-3}

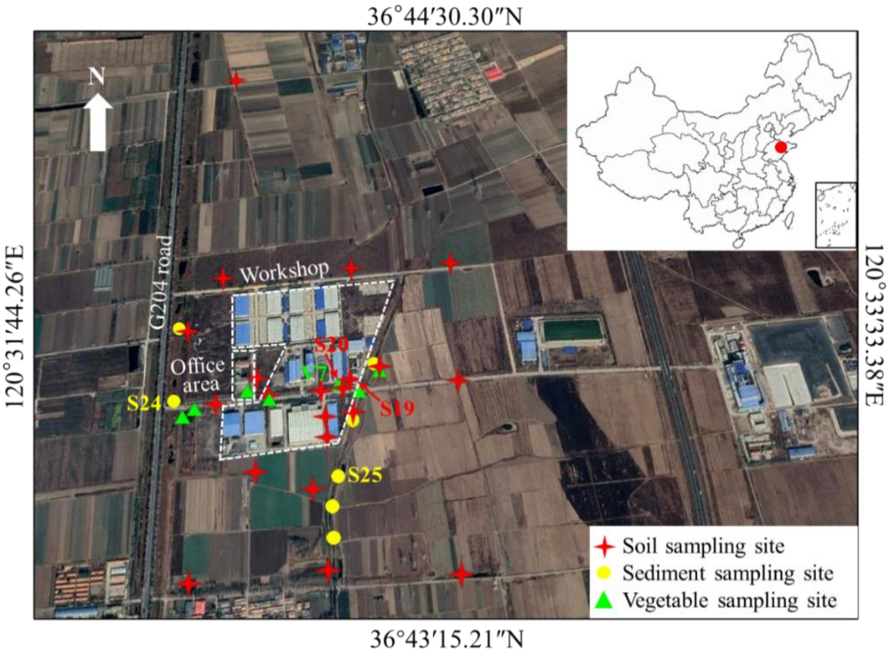

Figure S1. Map of the soil, sediment, vegetable sampling sites in regulated e-waste recycling site.

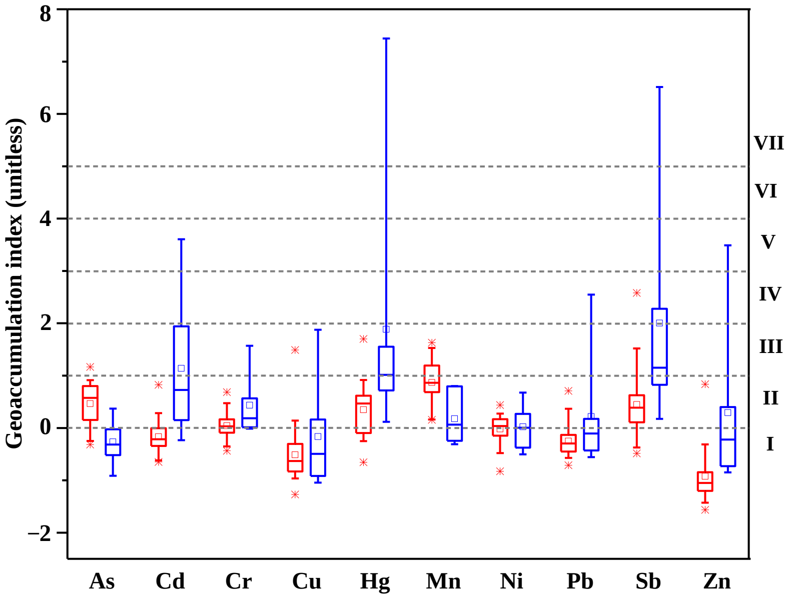

Figure S2. Box plots of the geoaccumulation index for heavy metals in soils (red) and sediments (blue) from regulated e-waste recycling site. Horizontal lines represent the 5%, 50%, and 95% values; the boxes represent the 25% and 75% values. The horizontal line and square in the box indicate the median and mean value; the asterisk below or above indicates the outlier value. Contamination classes: unpolluted ($I_{geo} \leq 0$) (I), unpolluted to moderately polluted ($0 < I_{geo} \leq 1$) (II), moderately polluted ($1 < I_{geo} \leq 2$) (III), moderately to heavily polluted ($2 < I_{geo} \leq 3$) (IV), heavily polluted ($3 < I_{geo} \leq 4$) (V), heavily to extremely polluted ($4 < I_{geo} \leq 5$) (VI), and extremely polluted ($I_{geo} > 5$) (VII), using the method developed by Muller [4].

References

1. Tang, Z.; Huang, Q.; Cheng, J.; Yang, Y.; Yang, J.; Guo, W.; Nie, Z.; Zeng, N.; Jin, L. Polybrominated Diphenyl Ethers in Soils, Sediments, and Human Hair in a Plastic Waste Recycling Area: A Neglected Heavily Polluted Area. *Environ. Sci. Technol.* **2014**, *48*, 1508–1516, doi:10.1021/es404905u.
2. Tang, Z.; Huang, Q.; Yang, Y.; Nie, Z.; Cheng, J.; Yang, J.; Wang, Y.; Chai, M. Polybrominated diphenyl ethers (PBDEs) and heavy metals in road dusts from a plastic waste recycling area in north China: implications for human health. *Environ. Sci. Pollut. Res.* **2015**, *23*, 625–637, doi:10.1007/s11356-015-5296-7.
3. Cheng, J.; Zhang, X.; Tang, Z.; Yang, Y.; Nie, Z.; Huang, Q. Concentrations and human health implications of heavy metals in market foods from a Chinese coal-mining city. *Environ. Toxicol. Pharmacol.* **2017**, *50*, 37–44, doi:10.1016/j.etap.2017.01.011.
4. Muller, G. Index of Geo-Accumulation in Sediments of the Rhine River. *GeoJournal*. **1969**, *2*, 108–118.
5. United States Environmental Protection Agency (USEPA). *Baseline Human Health Risk Assessment Vasquez Boulevard and I-70 superfund site Denver*; Denver (Co), 2001. Available online: https://www.epa.gov/sites/production/files/documents/hhra_vbi70-ou1.pdf (accessed on 31 January 2021).
6. Beijing Municipal Bureau of Quality and Technical Supervision (BMBQTS). Environmental site assessment guideline (DB11/T 656–2009); BMBQTS, Beijing. Available online: https://www.baidu.com/link?url=V_DFtVvt6IPOY1rE8yRzPcRze-thX5Iehk2IH-dQwQaJjH1eiAyXEoLMjpbms94iS-1Izhtj_Fcjk7wOIORT9VKt9wtu3WtMYWfv7tuTO5I-hj3xHk_NcnwSI2FjD2KVHYRC2I6meKP9RGI_3-Bks4VyEYqqI1xLR-QaFeCxldTau7o6h4rX2prhW98uGe0X3rygS9Wqp_yBUUmFAAStpgwLkZlkudFA7gP0KMR-2x-yw0D-MOziCKHN0yiXGFmSGB&wd=&eqid=af84f9d00001ea5d00000003606f0895 (accessed on 8 April 2021). (In Chinese)
7. Smith, R.L. Use of Monte Carlo Simulation for Human Exposure Assessment at a Superfund Site. *Risk Anal.* **1994**, *14*, 433–439, doi:10.1111/j.1539-6924.1994.tb00261.x.
8. United States Environmental Protection Agency (USEPA). *Risk Assessment Guidance for Superfund (EPA/540/1-89/002). Human Health Evaluation Manual (Part A)*; Office of Emergency and Remedial Response: Washington, DC, USA, 1989. Available online: https://www.epa.gov/sites/production/files/2015-09/documents/rags_a.pdf (accessed on 31 January 2021).
9. United States Environmental Protection Agency (USEPA). *Risk Assessment Guidance for Superfund: Volume III-Part A, Process for Conducting Probabilistic Risk Assessment*; Washington, DC, 2001. Available online: https://www.epa.gov/sites/production/files/2015-09/documents/rags3adt_complete.pdf (accessed on 31 January 2021).
10. He, A.; Li, X.; Ai, Y.; Li, X.; Li, X.; Zhang, Y.; Gao, Y.; Liu, B.; Zhang, X.; Zhang, M. Potentially toxic metals and the risk to children's health in a coal mining city: An investigation of soil and dust levels, bioaccessibility and blood lead levels. *Environ. Int.* **2020**, *141*, 105788, doi:10.1016/j.envint.2020.105788.
11. Health Canada (HC). *Federal Contaminated Site Risk Assessment in Canada-Part II: Health Canada Toxicological Reference Values (TRVs) and Chemical-Specific Factors*; Ottawa, Canada, 2004. Available online: http://publications.gc.ca/site/eng/387683/publication.html (accessed on 8 April 2021).
12. Integrated Risk Information System (USEPA IRIS). *2,2′,4,4′-Tetrabromodiphenyl ether (BDE-47) (CASRN 5436-43-1)*; Washington, DC: US Environmental Protection Agency, 2008. Available online: https://cfpub.epa.gov/ncea/iris/iris_documents/documents/toxreviews/1010tr.pdf (accessed on 31 January 2021).
13. Integrated Risk Information System (USEPA IRIS). *2,2′,4,4′,5-Pentabromodiphenyl ether (BDE-99) (CASRN 60348-60-9)*; Washington, DC: US Environmental Protection Agency, 2008. Available online: https://cfpub.epa.gov/ncea/iris/iris_documents/documents/subst/1008_summary.pdf (accessed on 31 January 2021).
14. Integrated Risk Information System (USEPA IRIS). *2,2′,4,4′,5,5′-Hexabromodiphenyl ether (BDE-153) (CASRN 68631-49-2)*; Washington, DC: US Environmental Protection Agency, 2008. Available online: https://cfpub.epa.gov/ncea/iris/iris_documents/documents/subst/1009_summary.pdf(accessed on 31 January 2021).
15. Integrated Risk Information System (USEPA IRIS). *2,2′,3,3′,4,4′,5,5′,6,6′-Decabromodiphenyl ether (BDE-209) (CASRN 1163-19-5)*; Washington, DC: US Environmental Protection Agency, 2008. Available online: https://cfpub.epa.gov/ncea/iris/iris_documents/documents/subst/0035_summary.pdf (accessed on 31 January 2021).
16. Alabi, O.A.; Bakare, A.A.; Xu, X.; Li, B.; Zhang, Y.; Huo, X. Comparative evaluation of environmental contamination and DNA damage induced by electronic-waste in Nigeria and China. *Sci. Total. Environ.* **2012**, *423*, 62–72, doi:10.1016/j.scitotenv.2012.01.056.
17. Leung, A.O.W.; Luksemburg, W.J.; Wong, A.S.; Wong, M.H. Spatial Distribution of Polybrominated Diphenyl Ethers and Polychlorinated Dibenzo-*p*-dioxins and Dibenzofurans in Soil and Combusted Residue at Guiyu, an Electronic Waste Recycling Site in Southeast China. *Environ. Sci. Technol.* **2007**, *41*, 2730–2737, doi:10.1021/es0625935.
18. Wu, Q.; Leung, J.Y.; Du, Y.; Kong, D.; Shi, Y.; Wang, Y.; Xiao, T. Trace metals in e-waste lead to serious health risk through consumption of rice growing near an abandoned e-waste recycling site: Comparisons with PBDEs and AHFRs. *Environ. Pollut.* **2019**, *247*, 46–54, doi:10.1016/j.envpol.2018.12.051.
19. Wu, X.F.; Tang, Z.W.; Shen, H.Y.; Huang, Q.F.; Tao, Y. Characterization of polybrominated diphenyl ethers (PBDEs) in dismantling and burning sites in electronic waste polluted area, south China. *Environ. Sci. Technol*. **2013**, *36*, 84–89. https://doi.org/10.3969/j.issn.1003-6504.2013.12.017. (In Chinese)

20. Wang, J.; Liu, L.; Wang, J.; Pan, B.; Fu, X.; Zhang, G.; Zhang, L.; Lin, K. Distribution of metals and brominated flame retardants (BFRs) in sediments, soils and plants from an informal e-waste dismantling site, South China. *Environ. Sci. Pollut. Res.* **2015**, *22*, 1020–1033, doi:10.1007/s11356-014-3399-1.
21. Ma, J.; Addink, R.; Yun, S.; Cheng, J.; Wang, W.; Kannan, K. Polybrominated Dibenzo-*p*-dioxins/Dibenzofurans and Polybrominated Diphenyl Ethers in Soil, Vegetation, Workshop-Floor Dust, and Electronic Shredder Residue from an Electronic Waste Recycling Facility and in Soils from a Chemical Industrial Complex in Eastern China. *Environ. Sci. Technol.* **2009**, *43*, 7350–7356, doi:10.1021/es901713u.
22. Wang, H.M.; Yu, Y.J.; Han, M.; Yang, S.-W.; Li, Q.; Yang, Y. Estimated PBDE and PBB Congeners in Soil from an Electronics Waste Disposal Site. *Bull. Environ. Contam. Toxicol.* **2009**, *83*, 789–793, doi:10.1007/s00128-009-9858-6.
23. Han, Y.; Cheng, J.; He, L.; Zhang, M.; Ren, S.; Sun, J.; Xing, X.; Tang, Z. Polybrominated diphenyl ethers in soils from Tianjin, North China: distribution, health risk, and temporal trends. *Environ. Geochem. Heal.* **2021**, *43*, 1177–1191, doi:10.1007/s10653-020-00645-9.
24. Jiang, Y.; Wang, X.; Zhu, K.; Wu, M.; Sheng, G.; Fu, J. Occurrence, compositional profiles and possible sources of polybrominated diphenyl ethers in urban soils of Shanghai, China. *Chemosphere* **2010**, *80*, 131–136, doi:10.1016/j.chemosphere.2010.04.007.
25. Li, W.L.; Ma, W.L.; Jia, H.L.; Hong, W.J.; Moon, H.B.; Nakata, H.; Minh, N.H.; Sinha, R.K.; Chi, K.H.; Kannan, K. Polybrominated Diphenyl Ethers (PBDEs) in Surface Soils across Five Asian Countries: Levels, Spatial Distribution, and Source Contribution. *Environ. Sci. Technol.* **2016**, *50*, 12779–12788, doi:10.1021/acs.est.6b04046.
26. Li, K.; Fu, S.; Yang, Z.Z.; Xu, X.B. Composition, Distribution and Characterization of Polybrominated Diphenyl Ethers (PBDEs) in the Soil in Taiyuan, China. *Bull. Environ. Contam. Toxicol.* **2008**, *81*, 588–593, doi:10.1007/s00128-008-9571-x.
27. Xiang, X.X.; Lu, Y.T.; Ruan, Q.Y.; Lai, C.; Sun, S.B.; Yao, H.; Zhang, Z.-S. Occurrence, Distribution, Source, and Health Risk Assessment of Polybrominated Diphenyl Ethers in Surface Soil from the Shen-Fu Region, Northeast China. *Environ. Sci.* **2020**, *41*, 368–376, doi:10.13227/j.hjkx.201907228. (In Chinese)
28. Wu, M.H.; Pei, J.C.; Zheng, M.; Tang, L.; Bao, Y.Y.; Xu, B.T.; Sun, R.; Sun, Y.F.; Xu, G.; Lei, J.Q. Polybrominated diphenyl ethers (PBDEs) in soil and outdoor dust from a multi-functional area of Shanghai: Levels, compositional profiles and interrelationships. *Chemosphere* **2015**, *118*, 87–95, doi:10.1016/j.chemosphere.2014.06.022.
29. Chen, Y.; Zhang, A.; Li, H.; Peng, Y.; Lou, X.; Liu, M.; Hu, J.; Liu, C.; Wei, B.; Jin, J. Concentrations and distributions of polybrominated diphenyl ethers (PBDEs) in surface soils and tree bark in Inner Mongolia, northern China, and the risks posed to humans. *Chemosphere* **2020**, *247*, 125950, doi:10.1016/j.chemosphere.2020.125950.
30. Jiang, Y.; Wang, X.; Zhu, K.; Wu, M.; Sheng, G.; Fu, J. Occurrence, compositional patterns, and possible sources of polybrominated diphenyl ethers in agricultural soil of Shanghai, China. *Chemosphere* **2012**, *89*, 936–943, doi:10.1016/j.chemosphere.2012.06.047.
31. Sun, J.; Pan, L.; Zhan, Y.; Lu, H.; Tsang, D.C.; Liu, W.; Wang, X.; Li, X.; Zhu, L. Contamination of phthalate esters, organochlorine pesticides and polybrominated diphenyl ethers in agricultural soils from the Yangtze River Delta of China. *Sci. Total. Environ.* **2016**, *544*, 670–676, doi:10.1016/j.scitotenv.2015.12.012.
32. Huang, C.; Zeng, Y.H.; Luo, X.; Ren, Z.; Tang, B.; Lu, Q.; Gao, S.; Wang, S.; Mai, B.X.X. In Situ Microbial Degradation of PBDEs in Sediments from an E-Waste Site as Revealed by Positive Matrix Factorization and Compound-Specific Stable Carbon Isotope Analysis. *Environ. Sci. Technol.* **2019**, *53*, 1928–1936, doi:10.1021/acs.est.8b06110.
33. Huang, Y.; Zhang, D.; Yang, Y.; Zeng, X.; Ran, Y. Distribution and partitioning of polybrominated diphenyl ethers in sediments from the Pearl River Delta and Guiyu, South China. *Environ. Pollut.* **2018**, *235*, 104–112, doi:10.1016/j.envpol.2017.12.049.
34. Luo, Q.; Cai, Z.W.; Wong, M.H. Polybrominated diphenyl ethers in fish and sediment from river polluted by electronic waste. *Sci. Total. Environ.* **2007**, *383*, 115–127, doi:10.1016/j.scitotenv.2007.05.009.
35. Fu, J. Study on the accumulation of dechorane plus: Compared with polybrominated diphenyl ethers in sediment from the Yongning River with an e-waste dismantling plant in Taizhou, Southeastern China. Master's Dissertation. Zhejiang University of Technology, Hangzhou, China, 2015. Available online: http://cdmd.cnki.com.cn/Article/CDMD-10337-1016036270.htm (accessed on 8 April 2021)(In Chinese)
36. Xiong, J.; An, T.; Zhang, C.; Li, G. Pollution profiles and risk assessment of PBDEs and phenolic brominated flame retardants in water environments within a typical electronic waste dismantling region. *Environ. Geochem. Heal.* **2015**, *37*, 457–473, doi:10.1007/s10653-014-9658-8.
37. Mai, B.; Chen, S.; Luo, X.; Yang, Q.; Sheng, G.; Peng, P.; Fu, A.J.; Zeng, E.Y. Distribution of Polybrominated Diphenyl Ethers in Sediments of the Pearl River Delta and Adjacent South China Sea. *Environ. Sci. Technol.* **2005**, *39*, 3521–3527, doi:10.1021/es048083x.
38. Zhang, T.; Yang, W.L.; Chen, S.J.; Shi, D.L.; Zhao, H.; Ding, Y.; Huang, Y.R.; Li, N.; Ren, Y.; Mai, B.X. Occurrence, sources, and ecological risks of PBDEs, PCBs, OCPs, and PAHs in surface sediments of the Yangtze River Delta city cluster, China. *Environ. Monit. Assess.* **2014**, *186*, 5285–5295, doi:10.1007/s10661-014-3777-7.
39. Li, F.; Zhang, H.; Meng, X.; Chen, L.; Yin, D. Contamination by persistent toxic substances in surface sediment of urban rivers in Chaohu City, China. *J. Environ. Sci.* **2012**, *24*, 1934–1941, doi:10.1016/s1001-0742(11)61033-4.
40. Yuan, X.; Wang, Y.; Tang, L.; Zhou, H.; Han, N.; Zhu, H.; Uchimiya, M. Spatial distribution, source analysis, and ecological risk assessment of PBDEs in river sediment around Taihu Lake, China. *Environ. Monit. Assess.* **2020**, *192*, 1–13, doi:10.1007/s10661-020-08286-2.

41. Da, C.; Wang, R.; Huang, Q.; Mao, J.; Xie, L.; Xue, C.; Zhang, L. Sediment Records of Polybrominated Diphenyl Ethers (PBDEs) from the Anhui Province Section of Yangtze River, China. *Bull. Environ. Contam. Toxicol.* **2021**, *106*, 334–341, doi:10.1007/s00128-020-03054-x.
42. Da, C.; Wang, R.; Ye, J.; Yang, S. Sediment records of polybrominated diphenyl ethers (PBDEs) in Huaihe River, China: Implications for historical production and household usage of PBDE-containing products. *Environ. Pollut.* **2019**, *254*, 112955, doi:10.1016/j.envpol.2019.07.123.
43. Sun, J.L.; Chen, Z.X.; Ni, H.G.; Zeng, H. PBDEs as indicator chemicals of urbanization along an urban/rural gradient in South China. *Chemosphere* **2013**, *92*, 471–476, doi:10.1016/j.chemosphere.2013.01.036.
44. Wen, S.; Yang, F.; Li, J.; Gong, Y.; Zhang, X.; Hui, Y.; Wu, Y.; Zhao, Y.; Xu, Y. Polychlorinated dibenzo-*p*-dioxin and dibenzofurans (PCDD/Fs), polybrominated diphenyl ethers (PBDEs), and polychlorinated biphenyls (PCBs) monitored by tree bark in an E-waste recycling area. *Chemosphere* **2009**, *74*, 981–987, doi:10.1016/j.chemosphere.2008.10.002.
45. Zhao, Y.X.; Qin, X.F.; Li, Y.; Liu, P.Y.; Tian, M.; Yan, S.S.; Qin, Z.F.; Xu, X.B.; Yang, Y.J. Diffusion of polybrominated diphenyl ether (PBDE) from an e-waste recycling area to the surrounding regions in Southeast China. *Chemosphere* **2009**, *76*, 1470–1476, doi:10.1016/j.chemosphere.2009.07.023.
46. Tian, M.; Chen, S.J.; Wang, J.; Luo, Y.; Luo, X.J.; Mai, B.X. Plant Uptake of Atmospheric Brominated Flame Retardants at an E-Waste Site in Southern China. *Environ. Sci. Technol.* **2012**, *46*, 2708–2714, doi:10.1021/es203669n.
47. Yang, Z.Z.; Zhao, X.R.; Zhao, Q.; Qin, Z.F.; Qin, X.F.; Xu, X.B.; Jin, Z.X.; Xu, C.X. Polybrominated Diphenyl Ethers in Leaves and Soil from Typical Electronic Waste Polluted Area in South China. *Bull. Environ. Contam. Toxicol.* **2008**, *80*, 340–344, doi:10.1007/s00128-008-9385-x.
48. Huang, H.; Zhang, S.; Christie, P. Plant uptake and dissipation of PBDEs in the soils of electronic waste recycling sites. *Environ. Pollut.* **2011**, *159*, 238–243, doi:10.1016/j.envpol.2010.08.034.
49. Wang, S.; Wang, Y.; Luo, C.; Li, J.; Yin, H.; Zhang, G. Plant selective uptake of halogenated flame retardants at an e-waste recycling site in southern China. *Environ. Pollut.* **2016**, *214*, 705–712, doi:10.1016/j.envpol.2016.04.071.
50. Wang, J.; Lin, Z.; Lin, K.; Wang, C.; Zhang, W.; Cui, C.; Lin, J.; Dong, Q.; Huang, C. Polybrominated diphenyl ethers in water, sediment, soil, and biological samples from different industrial areas in Zhejiang, China. *J. Hazard. Mater.* **2011**, *197*, 211–219, doi:10.1016/j.jhazmat.2011.09.078.
51. Jia, H.H.; Wang, X.T.; Cheng, H.X.; Zhou, Y.; Fu, R. Pine needles as biomonitors of polybrominated diphenyl ethers and emerging flame retardants in the atmosphere of Shanghai, China: occurrence, spatial distributions, and possible sources. *Environ. Sci. Pollut. Res.* **2019**, *26*, 12171–12180, doi:10.1007/s11356-019-04558-8.
52. Shi, S.X.; Zeng, L.Z.; Zhou, L.; Zhang, L.F.; Zhang, T.; Dong, L.; Huang, Y.R. Polybrominated diphenyl ethers in camphor bark from speedy developing urban in Jiangsu Province. *Environ. Sci.* **2011**, *32*, 2654–2660. (In Chinese)
53. Wu, Q.; Du, Y.; Huang, Z.; Gu, J.; Leung, J.Y.; Mai, B.; Xiao, T.; Liu, W.; Fu, J. Vertical profile of soil/sediment pollution and microbial community change by e-waste recycling operation. *Sci. Total. Environ.* **2019**, *669*, 1001–1010, doi:10.1016/j.scitotenv.2019.03.178.
54. Luo, C.; Liu, C.; Wang, Y.; Liu, X.; Li, F.; Zhang, G.; Li, X. Heavy metal contamination in soils and vegetables near an e-waste processing site, south China. *J. Hazard. Mater.* **2011**, *186*, 481–490, doi:10.1016/j.jhazmat.2010.11.024.
55. Luo, Y.; Yu, X.; Yang, Z.; Yuan, J.; Mai, B. Studies on heavy metal contamination by improper handling of e-waste and its environmental risk evaluation I. Heavy metal contamination in e-waste open burning sites. Asian, *J. Ecotoxicol.* **2008**, *3*, 34–41. Available online: https://en.cnki.com.cn/Article_en/CJFDTOTAL-STDL200801008.htm (accessed on 8 April 2021). (In Chinese)
56. Luo, Y.; Luo, X.; Yang, Z.; Yu, X.; Yuan, J.; Chen, S.; Mai, B. Studies on heavy metal contamination by improper handling of e-waste and its environmental risk evaluation. II. Heavy metal contamination in surface soils on e-waste disassembling workshops within villages and the adjacent agricultural soils. Asian, *J. Ecotoxicol.* **2008**, *3*, 123–129. Available online: https://xueshu.baidu.com/usercenter/paper/show?paperid=97bb4a53b576730d0e9eec5d88acfda0&site=xueshu_se (accessed on 8 April 2021). (In Chinese)
57. Tang, X.; Shen, C.; Shi, D.; Cheema, S.A.; Khan, M.I.; Zhang, C.; Chen, Y. Heavy metal and persistent organic compound contamination in soil from Wenling: An emerging e-waste recycling city in Taizhou area, China. *J. Hazard. Mater.* **2010**, *173*, 653–660, doi:10.1016/j.jhazmat.2009.08.134.
58. Wu, Z.; Zhang, L.; Xia, T.; Jia, X.; Wang, S. Heavy metal pollution and human health risk assessment at mercury smelting sites in Wanshan district of Guizhou Province, China. *RSC Adv.* **2020**, *10*, 23066–23079, doi:10.1039/d0ra01004a.
59. Qing, X.; Yutong, Z.; Shenggao, L. Assessment of heavy metal pollution and human health risk in urban soils of steel industrial city (Anshan), Liaoning, Northeast China. *Ecotoxicol. Environ. Saf.* **2015**, *120*, 377–385, doi:10.1016/j.ecoenv.2015.06.019.
60. Wang, S.; Cai, L.M.; Wen, H.H.; Luo, J.; Wang, Q.S.; Liu, X. Spatial distribution and source apportionment of heavy metals in soil from a typical county-level city of Guangdong Province, China. *Sci. Total. Environ.* **2019**, *655*, 92–101, doi:10.1016/j.scitotenv.2018.11.244.
61. Zheng, Y.M.; Chen, T.B.; He, J.Z. Multivariate geostatistical analysis of heavy metals in topsoils from Beijing, China. *J. Soils Sediments* **2007**, *8*, 51–58, doi:10.1065/jss2007.08.245.
62. Zhang, M.K.; Ke, Z.X. Heavy metals, phosphorus and some other elements in urban soils of Hangzhou city, China. *Pedosphere* ***2004****, 14, 177–185.*
63. Shi, G.; Chen, Z.; Xu, S.; Zhang, J.; Wang, L.; Bi, C.; Teng, J. Potentially toxic metal contamination of urban soils and roadside dust in Shanghai, China. *Environ. Pollut.* **2008**, *156*, 251–260, doi:10.1016/j.envpol.2008.02.027.

64. Luo, X.S.; Yu, S.; Zhu, Y.G.; Li, X.D. Trace metal contamination in urban soils of China. *Sci. Total. Environ.* **2012**, *421-422*, 17–30, doi:10.1016/j.scitotenv.2011.04.020.
65. Xia, X.; Yang, Z.; Cui, Y.; Li, Y.; Hou, Q.; Yu, T. Soil heavy metal concentrations and their typical input and output fluxes on the southern Song-nen Plain, Heilongjiang Province, China. *J. Geochem. Explor.* **2014**, *139*, 85–96, doi:10.1016/j.gexplo.2013.06.008.
66. Yang, P.; Mao, R.; Shao, H.; Gao, Y. An investigation on the distribution of eight hazardous heavy metals in the suburban farmland of China. *J. Hazard. Mater.* **2009**, *167*, 1246–1251, doi:10.1016/j.jhazmat.2009.01.127.
67. Zheng, Y.P. On Heavy metal pollution status and its assessment for the farmland soil of Henan Province. *J. Environ. Manage. Coll. Chin.* **2007**, *3*, 44–46,doi:10.13358/j.issn.1008-813x.2007.03.010. (In Chinese)
68. Geng, J.M.; Wang, W.B.; Wen, C.P.;Yi, Z.Y.; Tang, S.M. Concentrations and distributions of selenium and heavy metals in Hainan paddy soil and assessment of ecological security. *Acta Ecol. Sin.* **2012**, *32*, 3477–3486, doi:10.5846/stxb201105210667.
69. Zhao, Y.; Wang, Z.; Sun, W.; Huang, B.; Shi, X.; Ji, J. Spatial interrelations and multi-scale sources of soil heavy metal variability in a typical urban–rural transition area in Yangtze River Delta region of China. *Geoderma* **2010**, *156*, 216–227, doi:10.1016/j.geoderma.2010.02.020.
70. Ni, D.; Fang, H.G.; Wang, L.J. Present situation of soil environmental quality and protection countermeasures in Ji County. *Till. Cultivation* **2012**, *2*, 36–37. https://doi.org/10.13605/j.cnki.52-1065/s.2012.02.004. (In Chinese)
71. Chai, Y.; Guo, J.; Chai, S.; Cai, J.; Xue, L.; Zhang, Q. Source identification of eight heavy metals in grassland soils by multivariate analysis from the Baicheng–Songyuan area, Jilin Province, Northeast China. *Chemosphere* **2015**, *134*, 67–75, doi:10.1016/j.chemosphere.2015.04.008.
72. Niu, L.; Yang, F.; Xu, C.; Yang, H.; Liu, W. Status of metal accumulation in farmland soils across China: From distribution to risk assessment. *Environ. Pollut.* **2013**, *176*, 55–62, doi:10.1016/j.envpol.2013.01.019.
73. Chen, H.; Teng, Y.; Lu, S.; Wang, Y.; Wang, J. Contamination features and health risk of soil heavy metals in China. *Sci. Total. Environ.* **2015**, *512–513*, 143–153, doi:10.1016/j.scitotenv.2015.01.025.
74. Wang, F.; Leung, A.; Wu, S.; Yang, M.; Wong, M. Chemical and ecotoxicological analyses of sediments and elutriates of contaminated rivers due to e-waste recycling activities using a diverse battery of bioassays. *Environ. Pollut.* **2009**, *157*, 2082–2090, doi:10.1016/j.envpol.2009.02.015.
75. Quan, S.X.; Yan, B.; Lei, C.; Yang, F.; Li, N.; Xiao, X.M.; Fu, J.M. Distribution of heavy metal pollution in sediments from an acid leaching site of e-waste. *Sci. Total. Environ.* **2014**, *499*, 349–355, doi:10.1016/j.scitotenv.2014.08.084.
76. Luo, Y.; Luo, X.; Yang, Z.; Yu, X.; Yuan, J.; Chen, S.; Mai, B. Studies on heavy metal contamination by improper handling of e-waste and its environmental risk evaluation. IV. Heavy metal contamination of sediments in a small scale valley impacted by e-waste treating activities. Asian, *J. Ecotoxicol*. **2008**, *3*, 343–349. Available online: http://www.cnki.com.cn/Article/CJFDTOTAL-STDL200804007.htm (accessed on 8 April 2021). (In Chinese)
77. Chen, L.; Yu, C.; Shen, C.; Zhang, C.; Liu, L.; Shen, K.; Tang, X.; Chen, Y. Study on adverse impact of e-waste disassembly on surface sediment in East China by chemical analysis and bioassays. *J. Soils Sediments* **2010**, *10*, 359–367, doi:10.1007/s11368-009-0176-8.
78. Du, Y.; Wu, Q.; Kong, D.; Shi, Y.; Huang, X.; Luo, D.; Chen, Z.; Xiao, T.; Leung, J.Y. Accumulation and translocation of heavy metals in water hyacinth: Maximising the use of green resources to remediate sites impacted by e-waste recycling activities. *Ecol. Indic.* **2020**, *115*, 106384, doi:10.1016/j.ecolind.2020.106384.
79. Lin, C.; He, M.; Li, Y.; Liu, S. Content, enrichment, and regional geochemical baseline of antimony in the estuarine sediment of the Daliao river system in China. *Geochem.* **2012**, *72*, 23–28, doi:10.1016/j.chemer.2012.01.007.
80. Yang, Z.; Wang, Y.; Shen, Z.; Niu, J.; Tang, Z. Distribution and speciation of heavy metals in sediments from the mainstream, tributaries, and lakes of the Yangtze River catchment of Wuhan, China. *J. Hazard. Mater.* **2009**, *166*, 1186–1194, doi:10.1016/j.jhazmat.2008.12.034.
81. Guan, Q.; Cai, A.; Wang, F.; Wang, L.; Wu, T.; Pan, B.; Song, N.; Li, F.; Lu, M. Heavy metals in the riverbed surface sediment of the Yellow River, China. *Environ. Sci. Pollut. Res.* **2016**, *23*, 24768–24780, doi:10.1007/s11356-016-7712-z.
82. Mao, L.; Liu, L.; Yan, N.; Li, F.; Tao, H.; Ye, H.; Wen, H. Factors controlling the accumulation and ecological risk of trace metal(loid)s in river sediments in agricultural field. *Chemosphere* **2020**, *243*, 125359, doi:10.1016/j.chemosphere.2019.125359.
83. Bo, L.; Wang, D.; Li, T.; Li, Y.; Zhang, G.; Wang, C.; Zhang, S. Accumulation and risk assessment of heavy metals in water, sediments, and aquatic organisms in rural rivers in the Taihu Lake region, China. *Environ. Sci. Pollut. Res.* **2014**, *22*, 6721–6731, doi:10.1007/s11356-014-3798-3.
84. Zhang, P.; Qin, C.; Hong, X.; Kang, G.; Qin, M.; Yang, D.; Pang, B.; Li, Y.; He, J.; Dick, R.P. Risk assessment and source analysis of soil heavy metal pollution from lower reaches of Yellow River irrigation in China. *Sci. Total. Environ.* **2018**, *633*, 1136–1147, doi:10.1016/j.scitotenv.2018.03.228.
85. Xia, W.; Wang, R.; Zhu, B.; Rudstam, L.G.; Liu, Y.; Xu, Y.; Xin, W.; Chen, Y. Heavy metal gradients from rural to urban lakes in central China. *Ecol. Process.* **2020**, *9*, 1–11, doi:10.1186/s13717-020-00251-8.
86. Zheng, J.; Chen, K.H.; Yan, X.; Chen, S.J.; Hu, G.C.; Peng, X.W.; Yuan, J.G.; Mai, B.X.; Yang, Z.Y. Heavy metals in food, house dust, and water from an e-waste recycling area in South China and the potential risk to human health. *Ecotoxicol. Environ. Saf.* **2013**, *96*, 205–212, doi:10.1016/j.ecoenv.2013.06.017.
87. Liang, J.; Fang, H.; Zhang, T.; Wang, X.; Liu, Y. Heavy metal in leaves of twelve plant species from seven different areas in Shanghai, China. *Urban For. Urban Green.* **2017**, *27*, 390–398, doi:10.1016/j.ufug.2017.03.006.

88. Bi, C.; Zhou, Y.; Chen, Z.; Jia, J.; Bao, X. Heavy metals and lead isotopes in soils, road dust and leafy vegetables and health risks via vegetable consumption in the industrial areas of Shanghai, China. *Sci. Total. Environ.* **2018**, *619-620*, 1349–1357, doi:10.1016/j.scitotenv.2017.11.177.
89. Zhang, Q.; Yu, R.; Fu, S.; Wu, Z.; Chen, H.Y.H.; Liu, H. Spatial heterogeneity of heavy metal contamination in soils and plants in Hefei, China. *Sci. Rep.* **2019**, *9*, 1049, doi:10.1038/s41598-018-36582-y.
90. Gao, Z.; Fu, W.; Zhang, M.; Zhao, K.; Tunney, H.; Guan, Y. Potentially hazardous metals contamination in soil-rice system and it's spatial variation in Shengzhou City, China. *J. Geochem. Explor.* **2016**, *167*, 62–69, doi:10.1016/j.gexplo.2016.05.006.
91. Zeng, L.; Zhou, F.; Zhang, X.; Qin, J.; Li, H. Distribution of heavy metals in soils and vegetables and health risk assessment in the vicinity of three contaminated sites in Guangdong Province, China. *Hum. Ecol. Risk Assessment: Int. J.* **2018**, *24*, 1901–1915, doi:10.1080/10807039.2018.1431043.
92. Luo, Y.; Zhao, X.; Liu, H.; Xu, T.; Li, X.; Johnson, D.; Huang, Y. Bioaccumulation of heavy metals in the lotus root of rural ponds in the middle reaches of the Yangtze River. *J. Soils Sediments* **2017**, *308*, 83–2565, doi:10.1007/s11368-017-1692-6.
